# Global patterns of phosphatase activity in natural soils

**DOI:** 10.1038/s41598-017-01418-8

**Published:** 2017-05-02

**Authors:** O. Margalef, J. Sardans, M. Fernández-Martínez, R. Molowny-Horas, I. A. Janssens, P. Ciais, D. Goll, A. Richter, M. Obersteiner, D. Asensio, J. Peñuelas

**Affiliations:** 1 CSIC, Global Ecology Unit CREAF-CSIC-UAB, Bellaterra, 08193 Catalonia, Spain; 2 CREAF, Cerdanyola del Vallès, 08193 Catalonia, Spain; 3 Department of Biology, University of Antwerp, Universiteitsplein 1, B‑2610 Wilrijk, Belgium; 4 Laboratoire des Sciences du Climat et de l’Environnement, IPSL-LSCE CEA CNRS UVSQ UPSaclay, 91190 Gif-sur-Yvette, France; 5 Department of Microbiology and Ecosystem Science, University of Vienna, Vienna, A‑1090 Austria; 6 International Institute for Applied Systems Analysis (IIASA), Ecosystem s Services and Management, Schlossplatz 1, A-2361 Laxenburg, Austria

## Abstract

Soil phosphatase levels strongly control the biotic pathways of phosphorus (P), an essential element for life, which is often limiting in terrestrial ecosystems. We investigated the influence of climatic and soil traits on phosphatase activity in terrestrial systems using metadata analysis from published studies. This is the first analysis of global measurements of phosphatase in natural soils. Our results suggest that organic P (P_org_), rather than available P, is the most important P fraction in predicting phosphatase activity. Structural equation modeling using soil total nitrogen (TN), mean annual precipitation, mean annual temperature, thermal amplitude and total soil carbon as most available predictor variables explained up to 50% of the spatial variance in phosphatase activity. In this analysis, P_org_ could not be tested and among the rest of available variables, TN was the most important factor explaining the observed spatial gradients in phosphatase activity. On the other hand, phosphatase activity was also found to be associated with climatic conditions and soil type across different biomes worldwide. The close association among different predictors like P_org_, TN and precipitation suggest that P recycling is driven by a broad scale pattern of ecosystem productivity capacity.

## Introduction

Phosphorus (P) is an essential element for energy transport, cellular structures and nucleic acids and is thus essential for life. The weathering of parental material^1^ and the input from atmospheric dust deposition^2^ are the two main P sources for ecosystems growing on old and weathered soils. P availabilities are nevertheless low in most terrestrial ecosystems due to continuous, albeit low, leaching and sorption, leading to gradual P-occlusion in secondary minerals. Occlusion can also be reached by the accumulation of P in recalcitrant organic molecules associated to the slow and passive fraction of soil organic matter^3^. Organisms can assimilate only dissolved phosphate and therefore, phosphatase activity plays a fundamental role in the transformation of P from soil organic matter into available forms^4^. Phosphatase enzymes are produced by bacteria, fungi and plant roots and serve to cleave a phosphate group from its substrates, transforming complex and sometime unavailable forms of organic P into assimilable phosphate. Thus, phosphatase production depends on a combination of P demand from plants and microbes, available organic P substrate and P limitation of the soil. The rhizosphere is a narrow region of the soil that is directly influenced by root and mycorrhiza secretions of phosphatase and other enzymes, and sustains dense populations of root-associated and free-living microorganisms. Therefore, soil contains large quantities of intracellular (in living microbial cells) and extracellular (secretions of living cells or dead cellular material) phosphatases. Phosphatases can furthermore be stabilized in the soil on surface-reactive particles (e.g. clay and iron or aluminum oxides). This geochemically immobilized and yet enzymatically active fraction accounts for the enzymatic activity exhibited by soil, even in the absence of living organisms.

The pathways and forms of P during long-term pedogenesis are qualitatively well known and were summarized in 1973 by the Walker and Syers model^1^ and successive reviews^5^. Natural weathering progressively decreases the amount of primary inorganic forms of P^6^ until ultimately organic and secondary minerals might become the only reservoirs of this nutrient. Organic P (P_org_) can represent up to 90% of the total P in some old weathered soils^7, 8^ and microbial P often makes up a significant fraction of total P_org_^9^. In these old strongly weathered soils, phosphatase activity becomes crucial to accelerate the cycling of P between soil organic matter and plant uptake, and is a good measure of the ability of ecosystems to counteract P limitation^5^. Plant elemental composition is directly associated with phylogeny but also with climatic factors and the rate of nutrient supply^10^. Evidence suggests that phosphatase activity has important ecological consequences and could be intimately related to plant productivity and the diversity of biomes. Soil phosphatase activities have thus been widely studied^11, 12^.

Many experiments using added fertilization, changed temperature and water availability or other disturbances under controlled conditions have been designed to identify the natural drivers of phosphatase activity. Marklein and Houlton^13^ evaluated the effects of nitrogen (N), P or NP fertilization in 34 studies and found that N fertilization increased and P fertilization decreased phosphatase activity across different biomes. The changes in phosphatase activity from NP fertilization depend on the proportions of N and P added, different biomes exhibiting different thresholds^13, 14^. Hydric stress can also be a driving factor for phosphatase activity. For example, a reduction of water availability by 21% at Mediterranean sites decreased phosphatase activity by 31–40%^15^. Phosphatase activities are further correlated with the amount of soil organic matter, with the composition of microbial communities^16^, soil depth and ecological succession stage^4, 17^. Soil disturbances, such as fire –and the consequent reduction of microbial biomass-^18, 19^ or deforestation, also negatively affect the activity of this enzyme.

The heterogeneity in time and space of the distribution of enzymes in soil^20^ has restricted most of the phosphatase research to plot scale studies or controlled experiments under manipulated conditions. Many of these experiments suggest that soil nutrients and climate influence phosphatase activity, but a comprehensive overview is needed to document if there are large scale patterns that drive phosphatase activity in different soils and ecosystems across the globe.

Global change is bringing new environmental conditions to our planet, which has no equivalent in geological history at least for the pace at which conditions are changing. Increasing levels of carbon dioxide^21^, the variability in precipitation and temperature and the impact of pollutants and land management are expected to induce important changes in the stoichiometry of carbon (C) and N relative to P in natural systems. The availability of atmospheric C for plants is increasing, and that of N is being doubled by human inputs to ecosystems^13^, but these changes have not been accompanied by proportionally increased inputs of P in unmanaged ecosystems^22, 23^. Thus, understanding the capacity to mineralize P is crucial to understanding future ecosystem production changes.

**Figure 1:**
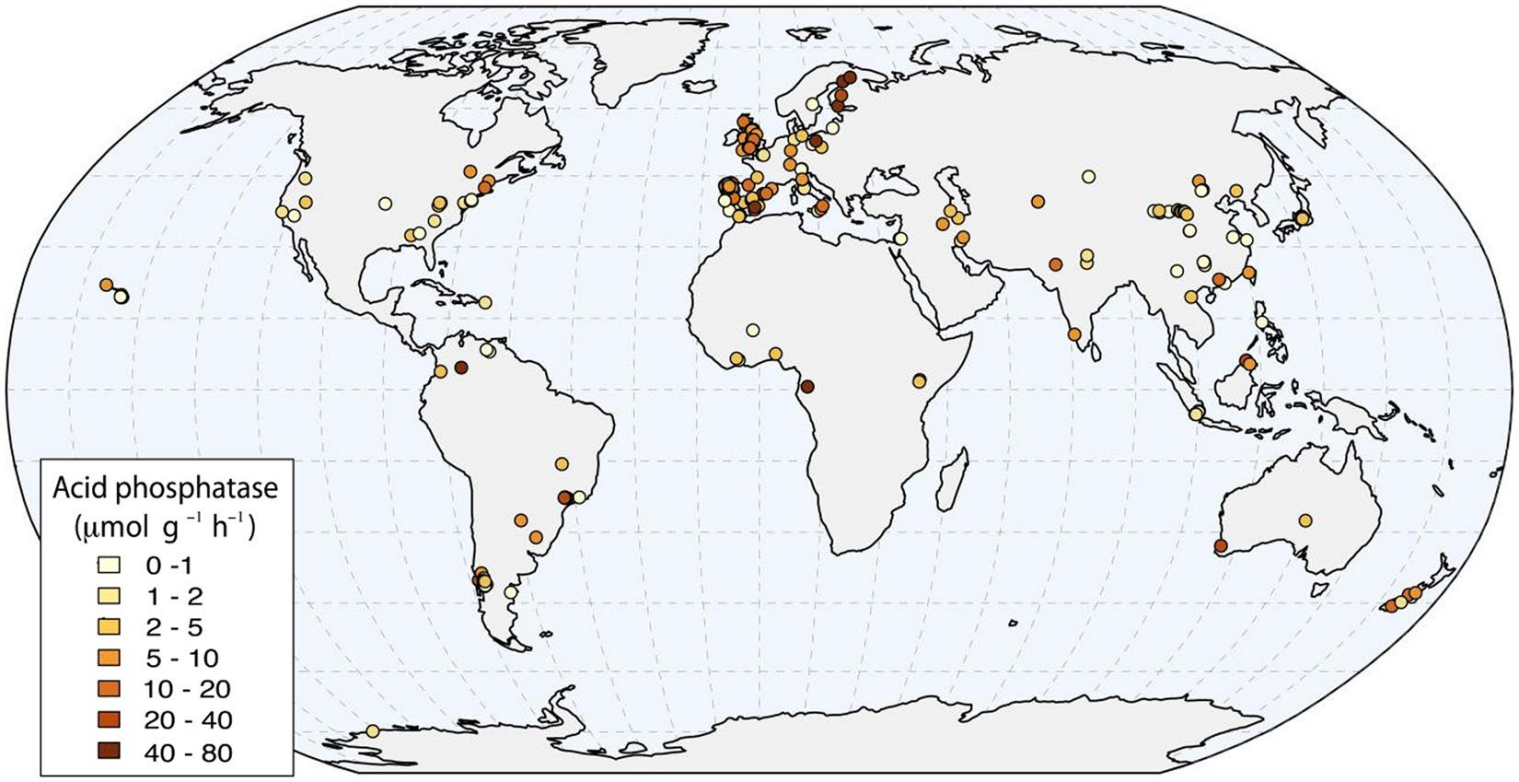
Distribution of the observations used in this review. A total of 378 sites around the world were obtained from 213 publications. This map has been created with R software^80^ (URL http://www.R-project.org) and rgdal^83^ and ggplot2^84^ packages, using free vector and raster map data from Natural Earth (URL http://www.naturalearthdata.com).

This study is the first compilation of phosphatase activities from natural systems that used comparable methodology, collecting 379 observations of acid (329) and alkaline (72) phosphatase activities from 183 studies (Fig. 1, Supplementary Information, Database References). In addition to phosphatase activity data, we collected: Total Carbon (TC), Total Nitrogen (TN), Mean Annual Precipitation (MAP), Mean Annual Temperature (MAT), Thermal Amplitude (AMP), P-Olsen, P-Bray, Resin-P, P_org_, Total Phosphorus (TP), Microbial Carbon (Micro C), pH and soil type and weathering status. We used this dataset to identify the climatic and soil variables associated with large-scale spatial gradients in soil phosphatase activity. A direct effect of climate forcing over soil and biome development and therefore over nutrient concentrations can be expected. In turn phosphatase activity has been largely related with not only phosphorus, but also nitrogen and carbon availability, as highlighted in this introduction. These premises have been used to elaborate the structural equation modeling, which will be detailed in the following sections. Our work aims to develop a first estimate of how phosphatase activity varies at global scale, which is needed for improving conceptual and numerical models describing soil P cycling and its repercussions on net primary productivity.

## Results

### Effects of climate and nutrient availability on phosphatase distribution

Site-level phosphatase activity data in our database ranged between 0.01 and 79 µmol g^−1^ h^−1^, with a mean of 11.6 ± 0.8 µmol g^−1^ h^−1^ (Fig. S1, *n* = 329) and were distributed along the seven continents (Fig. 1).

**Figure 2:**
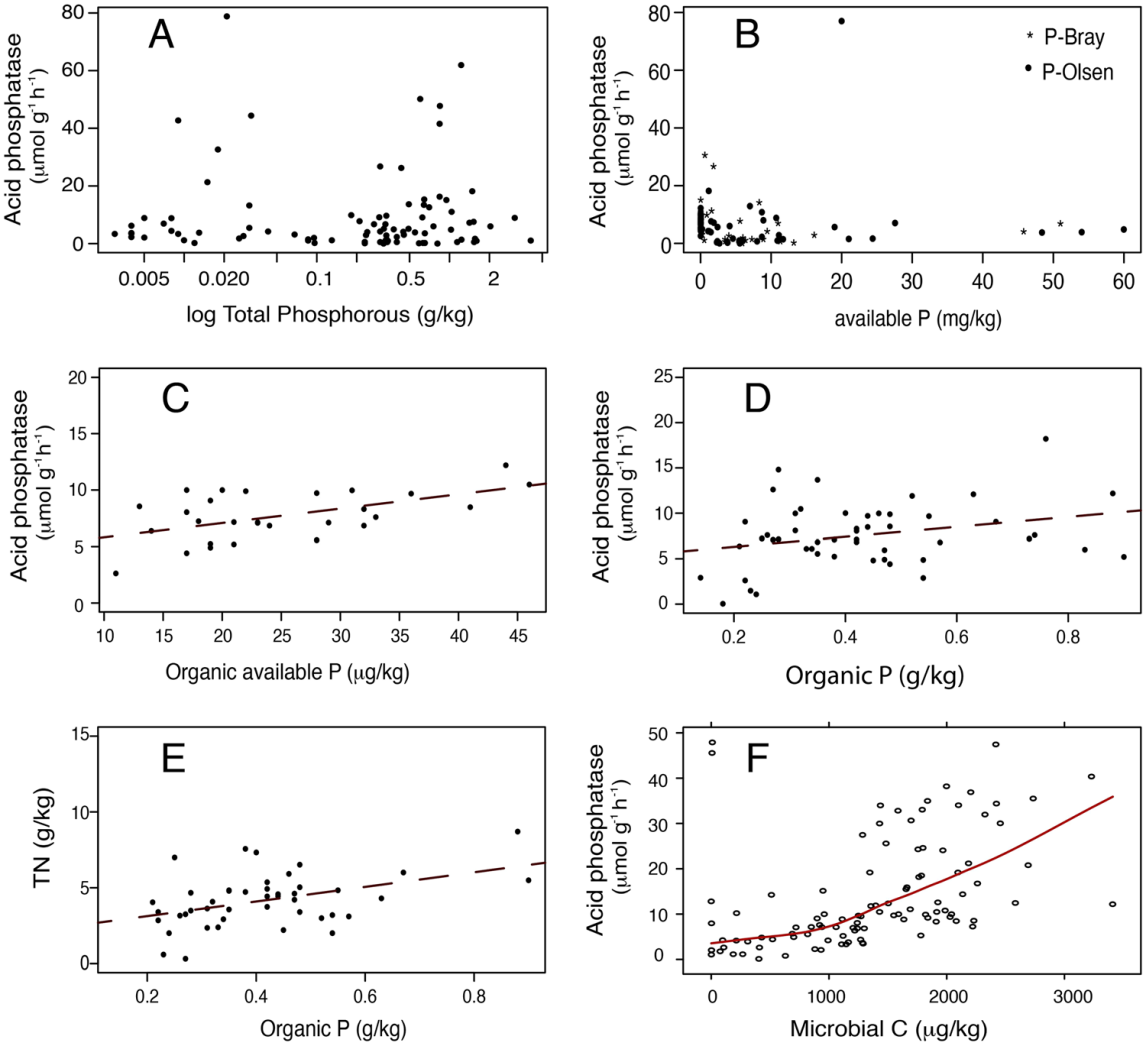
Acid phosphatase relationships. Acid phosphatase as a function of Total Phosphorus (**A**), Available P (**B**), organic available P (**C**) and organic P (**D**). Relationship between organic phosphorus and TN (**E**) and between acid phosphatase and Microbial C (**F**) are also shown for the mineral soils of our database.

Neither Total Phosphorus (TP) nor available P (P-Olsen, PBray or resin-P) was significantly correlated with acid phosphatase (Table 1, Fig. 2A,B). P-Olsen was positively correlated with alkaline phosphatase (R^2^ = 0.548, *p* < 0.01). P_org_ and organic available P (av P_org_) are the compiled phosphorus fraction that better correlate with acid phosphatase activity (Fig. 2C,D) and with other soil and climatic variables (Table 1). Total P_org_ correlates positively with acid phosphatase (Fig. 2C, R^2^ = 0.285, *p* < 0.01)), Total Carbon (TC, R^2^ = 0.502 *p* < 0.001), Microbial Carbon (MicroC, R^2^ = 0.419, *p* < 0.01), Total Nitrogen (TN, R^2^ = 0.436, *p* < 0.001), Mean Annual Precipitation (MAP, R^2^ = 0.492, *p* < 0.0001) and Thermal amplitude (AMP, R^2^ = 0.267, *p* < 0.01). Available P_org_ also strongly correlates with Acid phosphatase (R^2^ = 0.527, *p* < 0.0001) and with pH (R^2^ = −0.634, *p* < 0.0001) (Table 1). On the other hand, P-Olsen negatively correlates with Microbial C (R^2^ = −0.368, *p* < 0.01) while P-Bray shows a positive correlation with pH (R^2^ = 0.459, *p* < 0.001) and mean annual temperature (MAT, R^2^ = 0.442, *p* < 0.01) but negative with MAP (R^2^ = −0.356, *p* < 0.01).

**Table 1:** Correlation coefficients (r values) for relationships among phosphorus labile and organic forms and phosphatase activities, soil traits and climatic variables from our database: Acid phosphatase (Ac. Pasa), alkaline phosphatase (Alk. Pasa), pH, total Carbon (TC), microbial Carbon (MicroC), total Nitrogen, (TN), Clay content (% Clay), Mean annual precipitation (MAP), mean annual temperature (MAT), thermal amplitude (AMP).

	Ac. Pasa	Alk. Pasa	pH	TC	MicroC	TN	% Clay	MAP	MAT	AMP
**TP**	−0.027	0.172	−0.14	0.21	0.15	−0.036	−0.126	0.26*	−0.228*	0.195*
**P**_**org**_	0.285*	−0.183	0.016	0.502**	0.419*	0.436**	0.878	0.492***	0.024	0.267*
**Pav org**	0.527**		−0.634***	−0.045	0.307	0.016		−0.063	−0.143	−0.298
**P**-**Olsen**	0.043	0.548*	−0.003	0.08	−0.368*	−0.167	0.426	0.114	0.29*	0.084
**P**-**Bray**	−0.105	0.176	0.459**	−0.318	−0.536	−0.165	0.168	−0.356*	0.442**	−0.115
**resin**-**P**	−0.185		0.172	−0.249		−0.08	0.064	−0.104	−0.06	0.21

Significance levels are highlighted by * for p < 0.01,**p < 0.001, ***p < 0.0001.

**Figure 3:**
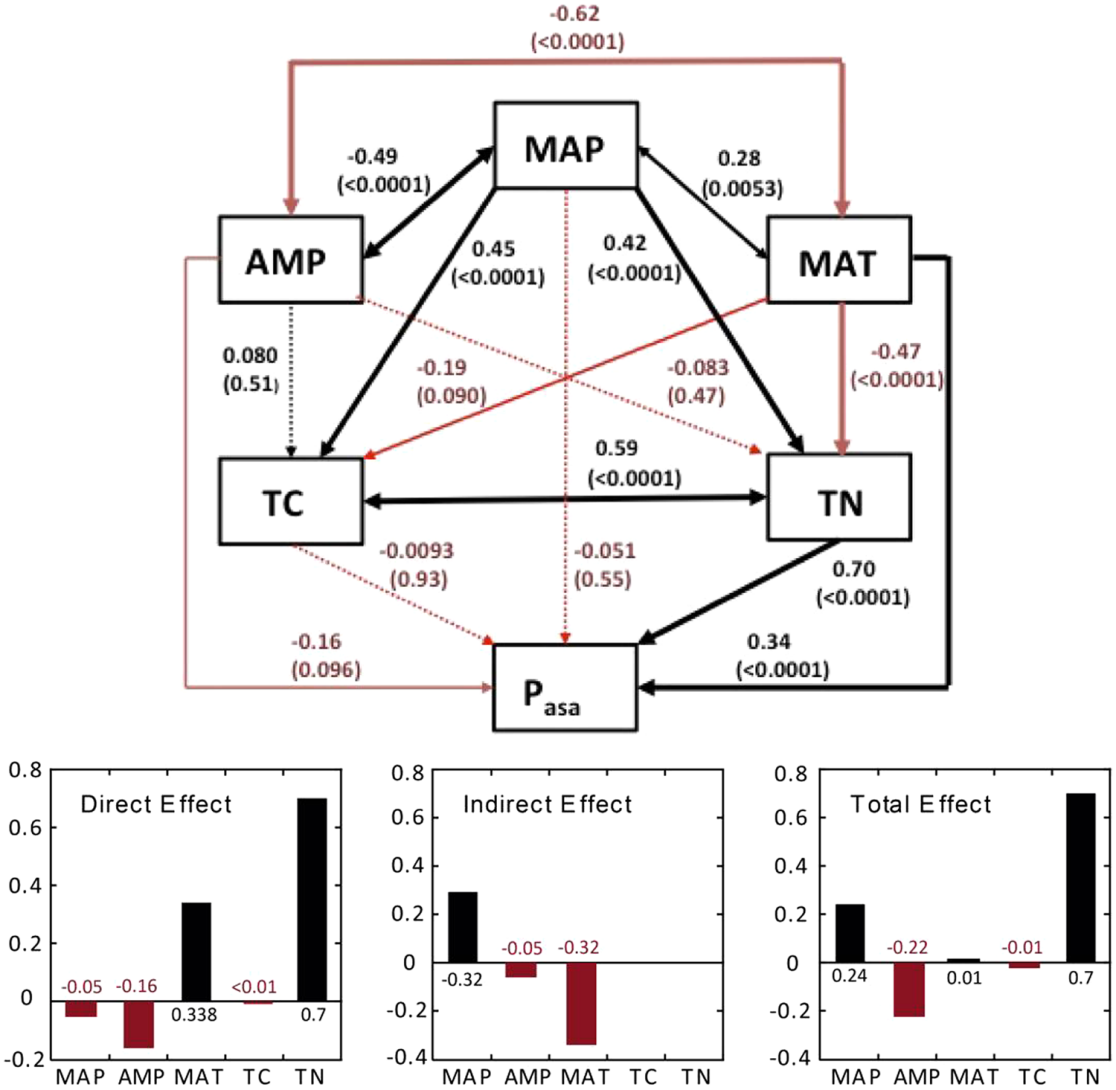
Structural equation modeling. The total effects of Total Nitrogen (TN) and Mean Annual Precipitation (MAP) were positive over phosphatase (Pasa). The effect of thermal amplitude (AMP) was negative, and Mean Annual Temperature (MAT) did not contribute significantly due to the opposite signals of its direct and indirect effects. Black arrows and bars represent positive effects, and red arrows and bars represent negative interactions. The width of the arrows is proportional to the amount of the variance explained and *P*-values are in brackets. *R* ^*2*^ for endogenous variables are TC = 0.18, Acid phosphatase = 0.5, TN = 0.28.

We then applied structural equation modeling (SEM, *R* ^2^ = 0.50) using log-transformed soil TN, MAP, MAT, TC and AMP was applied to elucidate the variables which statistically explain the gradients of phosphatase activity across sites. TP and different forms of available P were not considered for the SEM modeling because they showed no correlation to acid phosphatase patterns (Table 1). In turn, P_org_ was excluded from the predictors due to the insufficient number of site-data gathered in our database. The SEM results show the role of TN (direct effect = 0.7 ± 0.1) in predicting the gradients of acid phosphatase across sites (Fig. 3). The total effect of MAP (0.24) and the total effect of TN were positive over acid phosphatase, the total effect of AMP (−0.22) was negative, and MAT did not significantly contribute due to its opposing direct and indirect effects (see Fig. 3, Total effects). More specifically, MAT negatively influenced TN (−0.5 ± 0.1) but has positive direct effect (0.3 ± 0.1) on phosphatase. The main contribution of MAP to phosphatase activity was indirect and positive by contributing to the explanation of Total Nitrogen and Carbon (0.4 ± 0.1 to TN, 0.4 ± 0.1 to TC).

**Figure 4:**
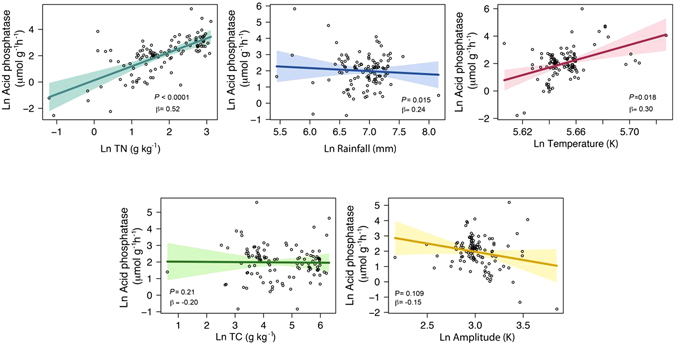
Partial residual plot. Partial residual plot of the variability of global Ln phosphatase activity (µmol g^−1^ h^−1^) explained by Ln TN (g kg^−1^), Ln MAP (mm), Ln MAT (K), Ln TC (g kg^−1^) and Ln AMP (K) (*visreg* R package^82^). Partial residual plots permit the evaluation of the effect of each variable on a full model without interactions. The linear model included TN, MAP, MAT, TC and AMP. Colorful areas indicate confidence band (0.95). All variables were Ln-transformed. See Supplementary Table 1 (Model 3) in the Supplementary Information for a summary of the linear model.

TN was a good predictor of acid phosphatase activity, better than P_org_ (Fig. 3), as shown by the rather high correlation between the log-transformed variables (Pearson correlation, 0.50, *p* < 0.0001). The more TN, the higher the phosphatase activity in the soil. Interactions among TN, MAP and MAT were quantified by a linear model that explained 26% of the variance (Model 1, Table S1) for all biomes combined without interactions among explanatory variables (n = 239, *R* ^2^ = 0.26, *p* < 0.0001) and 28% of the variance (Model 2, Table S1) for a model with interactions (n = 329, *R* ^2^ = 0.28, *p* < 0.0001). Modeling restricted to temperate sites (where the data are more abundant) with same predictors present a higher R^2^ (n = 104, *R* ^2^ = 0.46, *p* < 0.0001 with interactions and n = 104, *R* ^2^ = 0.41, *p* < 0.0001 without (Models 1 and 2, Table S2). Mild (MAT of 10–20 °C) and humid (MAP of 1000–3000 mm) climates had higher values of phosphatase activity. A more complex model including acid phosphatase activity data from all biomes and adding mean AMP and TC produced an *R* ^2^ of 0.48 without interactions (n = 110, *p* < 0.0001, Model 3, Table S2), indicating an influence of the amount of soil organic matter, seasonality and continentality (Fig. 4). A model with the same explanatory variables but including interactions, explained 62% of the variance (n = 110, *p* < 0.0001, Model 4, Table S1). Modeling phosphatase activity with 5 variables for temperate sites explained up to 83% of the cross-sites variance when including their interactions (*R* ^2^ = 0.83, n = 79, *p* < 0.0001) and 50% excluding interactions (*R* ^2^ = 0.50, n = 104, *p* < 0.0001, Models 3 and 4, Table S2).

Although this work is centered on acid phosphatase activity, the results of modeling alkaline phosphatase activity in relation to TN, MAT, MAP and pH are presented in Table S3 (R^2^ = 0.43, n = 67, *p* < 0.0001). Original pH of the soil was a significant explanatory variable for alkaline phosphatase, unlike in the modeling of acid phosphatase.

### Effects of soil weathering, community and forest type on phosphatase activity

**Figure 5:**
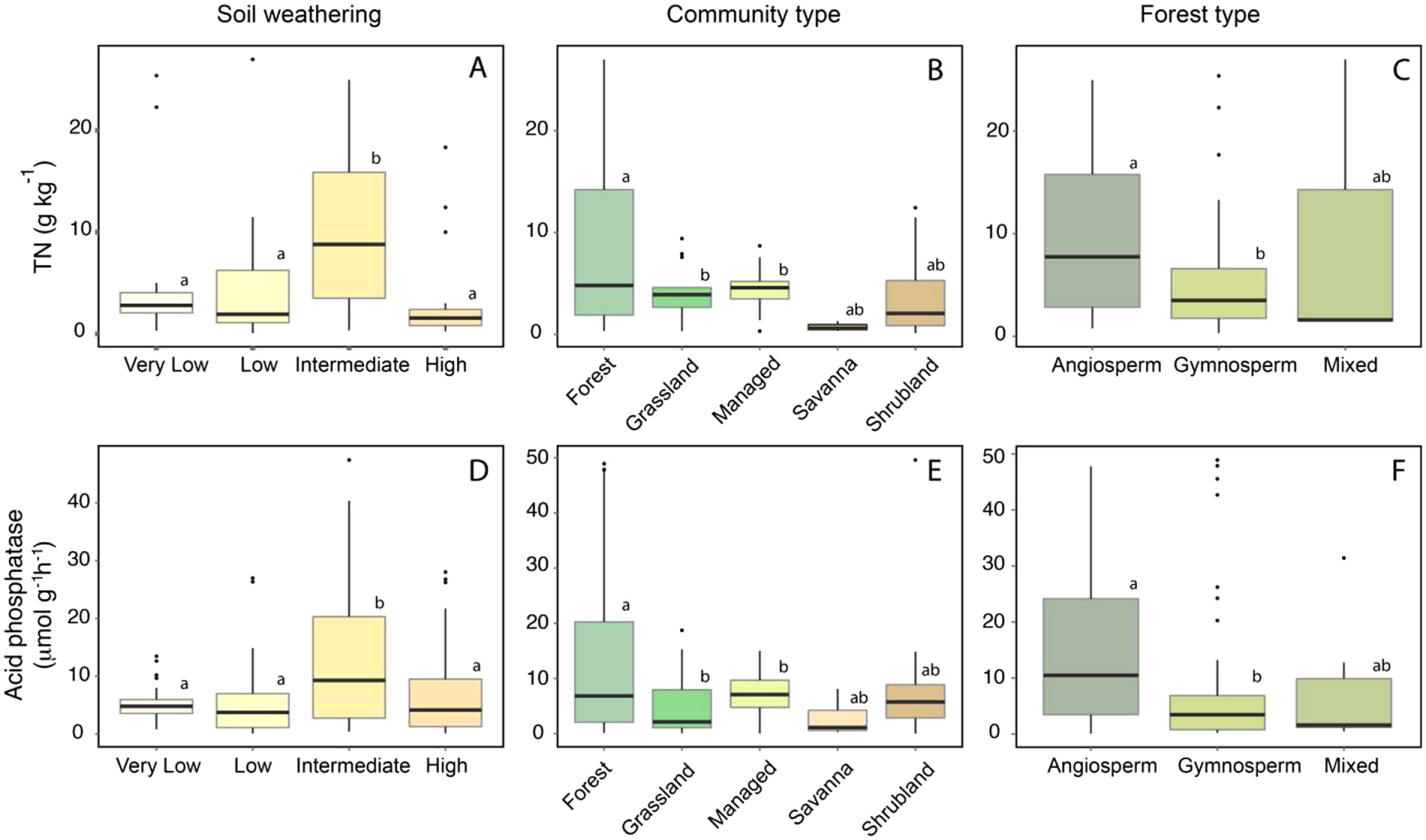
Acid phosphatase activity on different Soil weathering, Community and Forest types. Dependence of TN (**A**) and phosphatase activity (**D**) on the amount of soil weathering. Only sites with information for soil type were included (n = 204). Dependence of TN (**B**) and phosphatase activity (**E**) on community type (n = 291). Dependence of TN (**C**) and phosphatase activity (**F**) on forest class. Only sites with an accurate description of the vegetation were included (n = 171). Boxplot show median values (solid horizontal line), 50^th^ percentile values (box outline), 90^th^ percentile values (whiskers), and outlier values. Letters represent the results of Tukey’s post-hoc comparisons of group means.

Acid phosphatase activity shows coherent patterns on other environmental constraints such as the degree of soil weathering status (*F* = 8.15, *p* = 3.91e^−15^, see Methodology for explanation of the weathering stage classification). Phosphatase activity in very low, low and intermediate weathered soils averaged 5.2, 10.4 and 13.5 µmol g^−1^ h^−1^, respectively, indicating that soils with an intermediately weathered status have higher phosphatase activity (Fig. 5D). The phosphatase activity declined on the highly weathered soils (10.7 µmol g^−1^ h^−1^) a pattern that paralleled TN in the four weathering states (Fig. 5A–C).

Soil acid phosphatase activity also differed significantly among communities (*F* = 4.76, *P* < 0.001) and forest types (*F* = 4.52, *p* < 0.01) (Fig. 5E,F). Forest soils exhibited higher phosphatase activities (average of 14 µmol g^−1^ h^−1^) compared to grasslands (7 µmol g^−1^ h^−1^), managed soil (see methodology for managed description) (8 µmol g^−1^ h^−1^) and shrubland and savanna soils (9 µmol g^−1^ h^−1^). The gradients in average phosphatase activity across biomes also match those of TN (9.2, 7.8, 5.3 and 4.4 g kg^−1^ of TN in forest, grassland, managed land and shrubland and savanna types, respectively). Differences were found between angiosperm and gymnosperm forests. Both phosphatase and TN concentrations were higher in the angiosperm (14.5 µmol g^−1^ h^−1^, 9.25 g kg^−1^ TN) than the gymnosperm (8.19 µmol g^−1^ h^−1^, 5.6 g kg^−1^ TN) forests.

## Discussion

### Phosphorus forms and phosphatase production

Total phosphorus was found to be a poor predictor of phosphatase activity because it comprises the phosphorus part of primary mineral and also occluded recalcitrant forms, both being reservoirs that cannot be substrate for phosphatase enzymatic activity (Table 1, Fig. 2A). Our explanations for this result are (1) total P comprises mineral forms that are excluded from the loop of the cycle of P between soils and plants and (2) total P includes inorganic forms also not available for plants. Phosphorus is present in soil in several organic and inorganic forms, and only a small fraction of P_org_ is susceptible to release available phosphate after phosphatase reaction^24^. Av. P (Bray, Olsen, resin-P) has been traditionally presented as a good proxy for P availability in ecosystems. However, av. P did not correlate with phosphatase activity (Table 1, Fig. 2B), indicating that av. P relates to the actual availability of P, but not to potential capacity of the system to release P with the help of phosphatases. Instead, it can be considered an instantaneous picture of the immediately available P. Available P results from the balance between plant and microbial sinks, and sources from organic matter, itself partly of controlled by phosphatase activity. Thus, phosphatase relate only to the source term, whereas available P is also controlled by the sink terms. No pattern emerges between av P and phosphatase from the data we collected. Nevertheless, soils with high av. P_org_ and P_org_ (Fig. 2C,D) arise as the most useful P fractions to predict potential phosphatase activity^25^. Organic fraction includes very recalcitrant and moderately labile forms, but as a natural potential substrate for phosphatase, it is a proper indicator of the capacity of the system to obtain labile phosphorus. This reinforces the idea that not only labile but also the moderately labile forms^24^ are needed to quantify nutrient constraints in a soil-plant system. Therefore, a combination of organic P reservoir and its access door (phosphatase activity), can be a better indicator of P cycling capacity of the system than the direct measure of available forms. Unfortunately, despite we aimed to test statistically the robustness of P_org_ as an indicator for acid phosphatase, the compilation availability of P fraction measurements was much lower in our database than for TC and TN. For this reason, P_org_ was excluded from the structural equation modeling. However, Table 1 shows how P_org_ was found to be correlated with TN (Table 1, R^2^ = 0.44, *p* < 0.001), TC (R^2^ = 0.50, *p* < 0.001) and also microbial carbon (R^2^ = 0.42, *p* < 0.01), both first included in the presented SEM. This clearly indicates that soils with higher nitrogen content also are wider reservoirs for P_org_, presumably because they accumulate more organic matter. The result of the SEM and the possible direct effects of TN and TC over phosphatase production are discussed in the following section. Nevertheless, TN and TC can also be considered good proxies of soil P_org_, reinforcing the idea that the organic fraction of soil P, might be a good an indicator of the potential activity of acid phosphatase.

### Effects of nitrogen content and climate on phosphatase activity

The main large scale patterns of acid phosphatase activity distribution are explained by climatic conditions and soil nutrient concentrations. Soil TN is a good predictor of phosphatase activity gradients, explaining approximately 50% of the phosphatase variance in the linear models that included three or five variables (Fig. 4, TN β coefficient 0.51) and which were applied to the complete global data set. Our analysis is in line with studies reporting the stimulation of phosphatase activity by N fertilization^13^. Microorganisms, and presumably plants, are thought to respond to elemental imbalances in their resources by producing of enzymes targeting the element in need^26^. P only becomes growth limiting when and where availability of other resources, e.g. that of N, are sufficient. Total Nitrogen has been considered an appropriate benchmark for determining N availability in natural and agricultural soils^27–31^. Since N is the main growth-limiting nutrient in many areas of the world, high N availability relative to P is required for organisms to start investing in the production of phosphatase, a protein, and therefore a N-rich molecule^32–34^. Hence, the observed pattern of increasing phosphatase activity with TN may arise from increased N investment by plants and microbes into P acquiring enzymes at higher N availability^35^. TN is often well correlated with microbial biomass and microbial C^36^, which in turn is related high acid phosphatase activity as is demonstrated in our database. These patterns could, however, also be partly dependent on microbial community composition, as bacteria are thought to be more competitive than fungi in high-N environments^9^.

Our analysis shows that the effect of N was modulated by climatic conditions. The SEM analysis identified in particular an important role of water supply (MAP) for the activity of phosphatase, with a direct but also a strong indirect effect on TN and TC (Fig. 3), because the sites with higher rates of precipitation also frequently had more TN. This conclusion is in line with most of the experiments studying enzymatic production under different levels of water availability, which showed lower phosphatase activity under drought conditions^37^. Some of these studies reported that soil drought decreased soil phosphatase activity, decreasing P mineralization and short-term available P and increasing the P content of litter^37^. A lack of water may be responsible for the extremely low levels of microbial biomass and phosphatase in arid and semiarid biomes. Areas with harsh climatic conditions have been associated with lower litter input, decomposability, microbial and plant biomass and enzymatic activity^38^. On the other hand, effects of the other end of the water availability spectrum, like those found on flooded areas (swamps, flooded forests) are highly depending on reducing conditions and P speciation due to water saturation^39^ (see section 3.2).

Our data shows how phosphatase is dependent on climate patterns, but also TN concentration in soil. Our results suggest the coupling of different time-scale processes on the control of phosphatase activity. On one hand, several studies have reported enhanced phosphatase production under N fertilization experiments, revealing a short-term control of the enzymatic production. On the other hand, where climatic conditions are favorable and productivity higher, plants and microbes have modulated the nutrient pools. It is known that the sum of organic P and occluded P becomes a larger P pool in soils with increasing weathering intensity^40^. In our database TN and organic P are also strongly correlated so we propose that the effect of TN over phosphatase activity is also strongly influenced by long-term ecosystem evolution.

Our SEM model also identified a positive direct effect of temperature (MAT), which stimulates productivity, on phosphatase activity (Fig. 3) but a negative indirect effect via TN (probably related with harsh conditions), yielding almost no total effect of MAT on phosphatase activity. Several studies on various types of biomes report increased plant productivity and microbial activity at higher temperatures^41^ or increased phosphatase activity with atmospheric warming trends^42^. In contrast, cold temperatures and retarded decomposition are thought to lower N availability^41^.

### Patterns of phosphatase activity in different biomes

Several studies have suggested that the relationships among N and P availability and phosphatase activity should hold across biomes^43^, but evidence was thus far lacking. The presence of N in soil is the main factor explaining phosphatase activity in temperate climates, but phosphatase activity was also found to be strongly reduced by harsh climatic conditions in other parts of the world. Phosphatase activity is low in alpine environments (database average of 1.4 µmol g^−1^ h^−1^), probably restricted by the low pedogenetic development in steep or stony high-altitude areas with elevated thermal amplitude and cold conditions (SEM AMP total effect was −0.2). A high P availability from direct apatite weathering on young soils could also lead to a little need for phosphatases. The low rates of precipitation in arid and semiarid areas were also associated with very low phosphatase activities (database average of 4.28 µmol g^−1^ h^−1^) in soils that are usually TN depleted^44^. Water availability is the main restriction in arid areas, but cold conditions can hamper production and decomposition in boreal areas. Stagnant boreal wetland soils can become extremely rich in organic matter, in which organic P is trapped and thus unavailable to both plants and microbes. This would explain the extremely high phosphatase activity (database average of 46.7 µmol g^−1^ h^−1^) found in some peatlands^45^ and would also indicate a stronger P than N limitation under such conditions, as N still can be channeled into enzyme production for P acquisition. Activity, however, can be very low in boreal areas with rocky soils, low weathering rates^46^ and very high N limitation.

Given the low nitrogen, low carbon and short growing season climatic conditions, mean annual phosphatase activity would be expected to be low in soils of Mediterranean ecosystems. Nonetheless, high activities have been reported by studies at Mediterranean sites^37^, and the average activity for this biome was quite high in this study (9.97 µmol g^−1^ h^−1^). Mediterranean ecosystems have strong seasonality, and most measurements were collected in the spring or autumn when soil temperatures and water conditions are optimal for productivity^42^ and therefore enzymatic activity may be higher than the yearly average. The enzymatic content of the upper soil layers can vary 2-fold among seasons in communities that suffer from severe seasonality, such as those in the Mediterranean Basin^37^. Another hypothesis is that high phosphatase values in the soils of Mediterranean ecosystems are related to their low P content^15, 47–49^. Also, more enzymes might be required because in these systems, sclerophyll woody vegetation represents an important fraction of the forest understory, which requires more energy to be degraded.

Phosphorus has been considered the main limiting nutrient in tropical ecosystems^50^, related to low P soil substrates. Our study suggests that phosphatase activity in tropical and subtropical ecosystems (database average of 8.8 µmol g^−1^ h^−1^) does strongly depend on TN content. Tropical and subtropical sites in our data set had low TN values (average of 3.77 g kg^−1^), as expected from old soils with elevate mineral content and a large N pool in stand biomass. In these sites with relatively poor soils, most important enzymatic activity occurs at the litter layer, promoting a very rapid nutrient cycling and fast mineralization rates. In addition, the reduced conditions common in some flooded tropical forests, though, can lead to the release of previously unavailable P by the dissolution of iron oxides capable of binding P^39^. This process can be compensated by the formation of ferrous phosphate and an increased sorption capacity of iron hydroxides^39^. The labile P pathways in these environments are closely associated with the Fe and S cycles.

Temperate sites had higher phosphatase activities (14.4 µmol g^−1^ h^−1^). These ecosystems are frequently not water limited and commonly have well-developed soil horizons and high nitrogen content due to high atmospheric deposition^22^. Similar activities have been reported for temperate climax forests (23.5 and 6.63 µmol g^−1^ h^−1^ in O and Ah horizons, respectively)^51^.

To explore the differences among biomes partial residuals plots have been plotted from multiregression models with interactions including TN, MAT, MAP and AMP (Figure S3, Table S5). Phosphatases were related with TN in temperate and tropical sites (Figure S3), as well as alpine and arid sites (not shown). The effect of TN is not the main factor explaining phosphatase activity variance in Mediterranean sites due to the propensity of water availability and temperature to constrain nutrient cycling of the system.

### Phosphatase activity and soil weathering

Our findings identified differences in phosphatase activity associated with the degree of soil weathering and ecosystem development. Phosphatase activity was highest in intermediately weathered soils in our dataset (Fig. 5D). This pattern parallels those for TN (Fig. 5A), Carbon (*F* = 61.0, *P* < 0.0001, Supplementary Fig. 3) and microbial biomass (*F* = 27.5, *P* < 0.0001, Fig. S2), suggesting a relationship between enzymatic activity, microbial biomass and nutrient status^16, 52^. Though chronosequences and weathering stages are not strictly comparable, both patterns of change share common trends. Differences among low and high weathering status are comparable to differences between young and old soils described in chronosequences. Allison *et al*.^53^ described higher N and C trough older stages of a chronosequence, but C, N and P levels decline in the very last phase, on the oldest soils^53^. Nevertheless, P content of the soil is depleted much faster than C, so that the C:P and N:P ratios constantly increase with soil age^53, 54^. Peltzer *et al*.^55^ summarized the expected changes in soil properties over time, including an initial increase and a later decrease in soil N and C contents. Microbial biomass progressively increases in first stages of Franz Joseph soil chronosequence, until becoming progressively the first pool of total biomass phosphorus (plant and microbial)^9^. This suggests an intense competition between plants and saprotrophic microbes for soil phosphorus in mature soils^9^. Lower phosphatase activity with N limitation has been shown at retrogressed sites on volcanic islands^56^ like in the highly weathered soils of our dataset, where a very important component of the enzymatic activity occurs on the litter layer before than the soil (Fig. 5D). Assessing the relative contributions of nutrient availability and alterations in soil microbiota to the rate of change is difficult^55^. A shift of microbial communities from bacterial to fungal dominance is common in N-limited acidic soils^9, 52^, and such a shift can become an important driver of phosphatase activity. Also, a positive feedback loop of intensifying nutrient limitation can occur: lower nutrient concentrations alter microbial communities, decrease decomposing activity and further intensify nutrient limitation.

### Effects of community and forest type

Phosphatase activity and TN also differed significantly among communities (Fig. 5B,E). Phosphatase activity was consistently higher in forests (12.4 µmol g^−1^ h^−1^) than in grasslands (4.83 µmol g^−1^ h^−1^) (even when they share soil weathering status), or in managed or disturbed land (6.72 µmol g^−1^ h^−1^). Wardle *et al*.^57^ reported that nutrient cycling and enzymatic activity were intimately linked in above and belowground biota. Grasslands are systems composed of plant species that rapidly cycle nutrients, which benefits bacterial dominance (more competitive under high-N conditions)^52^. The control of plant composition over decomposers is context-dependent, but a higher proportion of fungi over bacteria are expected in forest soils. Root symbiotic mycorrhizal fungi are efficient producers of phosphatase^58^ and exert an important control over nutrient uptake^59, 60^. This is because of the large surface area that mycorrhiza develops in contact with the soil, interacting more efficiently with the mineral and organic surroundings^61^. The abundances of mycorrhiza types in soil are dependent on the distribution of their host species and grassland and forest soils exhibit different diversity of mychorrhiza^59, 62^, which may contribute to the final phosphatase production. The coexistence of different tree species produces a patchy microbial distribution in forests by the “single-tree” effects^54^ on the variability of the microbial communities. Soil communities, including root feeders and soil-engineering organisms, also determine the final specific enzymatic profiles. A good example are the epigeic earthworms and termites, whose mutualistic relationships can contribute to higher phosphatase activity^63^. However, we observe that despite forest and grassland soils showing differences in nutrient load capacity and primary productivity, a common pattern between TN and Acid Phosphatase underlies those disparities (Figure S4).

Information from the forest communities gathered in our data set allowed us to identify differences between angiosperm and gymnosperm soils (Fig. 5F). Soil phosphatase was significantly higher in the angiosperm forests (14.5 vs 8.2 µmol g^−1^ h^−1^) compared to gymnosperm ones. This pattern was consistent for the Mediterranean and temperate biomes but not the tropical or subtropical sites where angiosperm dominance did not allow this comparison. A disparity between these two tree classes would suggest that in forests a significant proportion of the phosphatase activity in soils may be produced by plant roots and mutualistic mychorrhiza. Lower biological activity could be expected in gymnosperm soils, which are better adapted to harsh conditions (water stress in Mediterranean forests and lower temperatures in boreal ones) in comparison with angiosperm forests. However, we did not identify large differences in rainfall between both groups of forest compared in our database, fact that suggests that enzymatic differences depend on biological differences rather than climatic ones. The higher phosphatase activity in angiosperm soil may be associated with TN values (Fig. 4C). Some studies highlighted that low Nitrogen Use Efficiency (NUE) appears to be a significant disadvantage for gymnosperm plants under N limitation^64^ that could prevent phosphatase exudation. Differences among microbial life, mycorrhiza dominances^65^ or stoichiometry observed between angiosperm and gymnosperm classes^66^ could be also behind these differences in phosphatase activity. Previous studies have reported higher tissue P contents in angiosperms than gymnosperms^66^, perhaps leading to a higher phosphatase requirement and thus higher enzyme activities. This differential demand of P arises as an important biological trait that could have driven both separate evolutionary paths.

### Implications for predicting the effects of global change on phosphatase activity

Many of Earth’s landscapes and ecosystems have been severely disturbed in recent decades. Together with local land management, global processes such as atmospheric CO_2_ enrichment or rising sea levels are transforming our world. The effects of future climate and environmental change on soil-P cycling and enzymatic activities are hard to predict, because the feedbacks among soil chemical properties and above- and belowground life are still poorly understood. Higher levels of atmospheric CO_2_ can stimulate phosphatase synthesis by changing litter quality and enhancing plant and microbial productivity^57, 67^, from which we could expect a faster turnover of P. Our data suggest that increasing temperatures could produce similar changes (Fig. 4), but also suggest that possible effects of CO_2_ and temperature can be constrained by water limitation.

Imminent aridification is predicted for the Mediterranean Basin, Central America and some areas of South America, southern Africa and Australia^68^. There is evidence that water limitation will strongly affect the stoichiometry of soil organic matter and various plant tissues. Drought has been related to decreases in soluble organic C and P due to lower microbial biomass and activity^42^ a pattern that is also seen at the arid and semiarid sites in our study. More available P_org_ is released during drought by higher production of litter in the short-term, but available inorganic P and phosphatase activity decrease, leading to strong mid- and long-term P limitation^43^. Moreover, positive feedbacks can accelerate P loss by rapid leaching of soluble P during re-wetting, by an osmotic shock from microbes or from the leaching of nutrient aggregates^30^. Increasing water shortage and a higher likelihood of extreme events may decrease phosphatase activity, inducing the degradation of some ecosystems and the gradual change to communities that are less dependent on water and nutrients (e.g. Mediterranean forest towards shrublands). This change can be interpreted as a positive feedback on climate change, because less C is captured as biomass, and the ecosystem can become a source of CO_2_. Most predictions of global change also account for an increase in the frequency of fire in various biomes, such as circumboreal forests (up to 50%)^69^ or Mediterranean countries^70^, with important impacts on the C budget. Various forms of P and phosphatase activity can be considerably lower after a fire^19^ and microbial biomass becomes the main factor accounting for the P status and recovery. In boreal forest, fire might increase the available P for the surviving and pioneer plants.

Global change is predicted to bring a higher input of N due to anthropogenic fertilization^13^. Our model (Fig. 3) predicts that phosphatase activities will be higher in ecosystems subject to atmospheric-N deposition. Enhancement of soil N availability, especially in poor-nutrient soils, is a common characteristic of invasive species, which will arise as an emerging driver of global change worldwide. Some systems that do not suffer from hydric stress are consequently expected to shift toward P limitation and higher phosphatase activity^71^.

Our models and literature compilation suggest an uncertain scenario for the coming years: the effects of higher CO_2_ levels, N fertilization and temperature are expected to increase phosphatase activity and the rate of turnover of P_org_ in soils, but only if enough water is available. In other areas, water restriction will induce P limitation and lower phosphatase activity, an effect that could be aggravated by recurrent fires.

This study is the first to date to analyze global patterns of phosphatase activity in soil. Soil TN is the main factor that we looked at, explaining spatial gradients of phosphatase activity at a global scale. Higher temperature and precipitation were further found to be positively associated with phosphatase activity. An important part of the effect of rainfall on phosphatase activity was indirect, occurring through the effect of water availability on soil TN. Where the climatic conditions are not specifically limiting -such as temperate and tropical forest-, soil N arises among the studied factors as the most determinant in limiting acid phosphatase activity. However, on other sites that can undergo temperature or water limitations, the main control over phosphatase activity would be exerted by climate –such as arid or Mediterranean areas-. Mutualistic interactions between microbes and plants can guarantee P cycling, but chemical fixation, release and effective use of phosphatase can be an ultimately constrain for P uptake. Soil geochemical conditions play an important role in determining phosphatase activity, and may be specifically important in tropical systems. This study identified some global and regional variables empirically associated with soil phosphatase content, but the complexity and importance of the issue merits a further combination of experimentation and wider data collection from natural soils (especially from boreal, alpine and arid sites). Such studies should include an analysis of the various P and N pools, including microbial and plant P and N for understanding the coupling of the N and P cycles as a mechanism arising from our study, by which organisms might be able to compensate for the anthropogenic disturbances in geochemical cycles. Future work is also required to determine the effects of global change on soils around the world for developing robust models of changes in the stoichiometric relationships for all types of ecosystems.

## Materials and Methods

### Data collection

We searched the ISI Web of Science using combinations of the following key words: acid, alkaline, activity, availability, available, bulk, carbon, concentration, content, C:K, C:N, C:P, enzyme, molybdenum, nitrogen, N:K, N:P, phosphatase, phosphorus, ratio, soil, solution, stoichiometrical, stoichiometric and stoichiometry (Supplementary Informations, Database References).

Acid phosphatase (phosphomonoesterase) activities in the selected publications were determined using disodium-*p*-nitrophenyl phosphate (pNPP) or disodium phenyl (Ph) phosphate as a substrate. In these studies, a dissolved solution with fresh soil was buffered (using citric, boric, maleic and hydrochloric acids) at pH 5–6.5 and incubated at 30–37 °C for 1–12 h (time and temperature depended on the assay). The reactions were stopped by adding CaCl_2_ and NaOH and filtered to prevent interference from possible precipitates. Phosphatase activity in all publications was obtained by spectrophotometric absorbance^11, 72–74^. Nannipieri *et al*.^12^ provided a good compilation of methodologies. The spectrophotometric absorbance method estimates the maximum potential activity of soil phosphatase, enabling useful comparison among studies, which will be reported as phosphatase activity in this study.

The diversity of phosphatase enzymes is an important hurdle for establishing the role of phosphatase in the phosphorus (P) cycle. Methodologies are unfortunately limited by only measuring a certain type of phosphatase or substrate at the time while acid and alkaline phosphatases usually coexist in the soil. Moreover, most studies measure only monophosphates, whereas both mono- and diester phosphates are cleaved by phosphatase^12^. Enzymatic activity is always measured in the laboratory, and P uptake by organisms cannot be strictly inferred by the maximum potential phosphatase activity^13^.

Although acid and alkaline phosphatases sometimes coexistbut dominate within different ranges of soil pH, we focused our analyses on acid phosphatase for being measured on pH more representative of natural soil conditions than the alkaline one. Alkaline phosphatase is typically measured at high pH, far away from standard natural soil pH values. Acid phosphatase is moreover the most extensively used phosphatase activity measurement and from which we have a more robust dataset.

For all observations, acid and alkaline phosphatase activity, information was collected for the location (latitude and longitude), soil type (FAO classification) and biome (classified as arid, boreal, Mediterranean, alpine, temperate and tropical, which included both tropical and subtropical biomes) from the original papers. Data for total nitrogen (TN), total carbon (TC), mean annual temperature (MAT), mean annual precipitation (MAP) and thermal amplitude (AMP) for each site were obtained from the original publications. Missing climatic information was provided using WorldClim^75^. The sites spanned latitudes from 74°S to 67.3°N and longitudes from 159.5°W to 172.73°E, with MAT varying between −2 and 34 °C, MAP between 120 and 7000 mm and AMP between 8.5 and 51.5 °C.

We obtained 72 measurements of alkaline phosphatase activity and 329 geo-referenced sites for acid phosphatase activity from 183 studies of 213 reviewed publications. From these, 50 measurements were from the Southern Hemisphere, and 279 were from the Northern Hemisphere (Fig. 1).

When there was enough information, samples were classified among general community types: forest (natural forest), grassland (natural grassland), shrubland (natural shrubland) and managed systems (including forest plantations, pastures, and managed meadows.

The soil type at each sampling site was included in the data set obtained from the original publications and followed the FAO soil classification^76^. For comparing the phosphatase and nutrient statuses of the soil, we classified the soil types based on the amount of weathering as very low (Leptosol, Regosol and Solonchak), low (Arenosol, Fluvisol, Cambisol and Calcisol), intermediate (Nitisol, Phaeozem, Luvisol, Plonthosol, Kastanozem, Solonetz, Gleysol, Andosol and Lixisol) and high (Acrisol, Ferralsol, Alisol and Podzol) weathering.

Determining the proportions of soil P pools and the avP or P_org_:TN ratio of soil is a key step for the understanding of nutrient coupling, because soil total P itself is not a good indicator of the biological availablity of this nutrient by the system. Insufficient information was unfortunately provided by most studies of enzymes involving the available forms of P, which prevented us from including this parameter in our analyses.

## Data analysis

A correlation matrix was performed to investigate the relationship of the different P forms with soil and climatic traits. This approach was complemented by scatterplots of the most significant relations. Regression models were calculated with R software^77^.

### Structural equation modeling

We used structural equation modeling (SEM) to analyze the factors explaining the maximum variability of phosphatase activity: MAP, MAT, AMP and soil TC and TN. This analysis provided a suitable way to stablish a graphical representation of multifactor direct and indirect effects on internal factors when complex relationships are expected among the independent variables on dependent variables. We fit the models using the sem R package^78^ and acquired the minimum adequate model using the Akaike information criterion. Standard errors and the significance levels of the direct, indirect and total effects were calculated by bootstrapping^79^ (1200 repetitions, Fig. 3).

### Phosphatase modeling

The response of acid phosphatase to changing conditions in the TN, MAP, MAT, TC and AMP predictors was modeled using the R stats package^80^. We previewed the data sets to determine the best method to model the dependence between response and predictors. We consequently log-transformed both the predictors and the response covariates, which also helped to ensure that the residuals were approximately normally distributed and homoscedastic. The Kolmogorov-Smirnov test has been performed to prove residuals normality^81^ (not shown).

A first model including worldwide biomes was developed to determine the relationships between the global patterns of phosphatase activity and nutrient availability (TN), climatic variables (MAT and MAP) with and without corresponding second-degree interactions (Models 1 and 2, Table S1). A more complex modeling scenario was next developed, including TC and AMP as additional predictor variables (Models 3 and 4, Table S1). The exercise was replicated using only the temperate sites (the biome that provided the most observations) to compare the behavior of phosphatase on a global scale and under more restricted climatic conditions (Models 1-4, Table S2). The modeling of alkaline phosphatase using TN, MAT, MAP and pH is presented also in the Supplementary Information (Table S3). In that case, the reduced number of samples prevented us from developing a model that also included TC as an explanatory variable. In all cases, significance level is considered at p < 0.05.

Partial residual plots of the global models were obtained using the *visreg* package^82^ to evaluate the effect of each variable on the full model without interactions (Fig. S3 and S4).

## Electronic supplementary material

Supplementary Information
