# Supplementary material for: Global patterns of phosphatase activity in natural soils
Source: Sci Rep. 2017 May 2;7:1337. doi: 10.1038/s41598-017-01418-8 (PMC5431046; doi:10.1038/s41598-017-01418-8)
Supplement: Supplementary file 1 — Supplementary Information [file 41598_2017_1418_MOESM1_ESM.pdf]

Global patterns of phosphatase activity in natural soils

Margalef, O.* [1,2]**; Sardans, J.** [1,2]**; Fernández-Martínez, M.** [1,2]**; Molowny-Horas, R.** [2]**; Janssens, I.A.**[3]**; Ciais, P.**[4]**; Goll, D.** [4]**, Richter, A.** [5]**; Obersteiner. M.**[6]**; Asensio, D.** [1,2]**; Peñuelas, J.**[1,2]

[1] CSIC, Global Ecology Unit CREAF-CSIC-UAB, Cerdanyola del Vallès, 08193 Catalonia, Spain.
[2] CREAF, Cerdanyola del Vallès, 08193 Catalonia, Spain.
[3] Research Group of Plant and Vegetation Ecology (PLECO), Department of Biology, University of Antwerp, B-2610 Wilrijk,
Belgium.
[4] Laboratoire des Sciences du Climat et de l'Environnement, IPSL, 91190 Gif-sur-Yvette, France.
[5] Department of Microbiology and Ecosystem Science, Division of Terrestrial Ecosystem Research, University of Vienna, Austria
[6] International Institute for Applied Systems Analysis (IIASA), Ecosystem s Services and Management, Schlossplatz 1, A-2361 Laxenburg, Austria.

SUPPLEMENTARY INFORMATION

Competing financial interests
The author(s) declare no competing financial interests

Fig S1 Histogram
Histogram of Acid phosphatase measurements compiled in these database (left, n=329). We log-transformed Acid phosphatase values for the modeling to ensure that the residuals were approximately normally distributed (right, n=329).

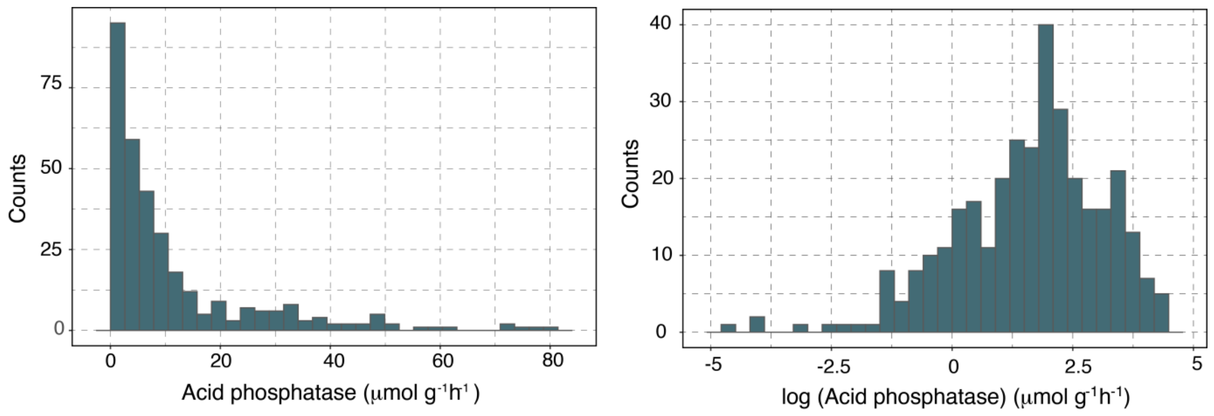

Fig S2 Soil weathering stages traits
Dependence of TC (A) and microbial C (B) on the amount of soil weathering (very low, low, intermediate and high). Boxplot show median values (solid horizontal line), 50th percentile values (box outline), 90th percentile values (whiskers), and outlier values. Letters represent the results of Tukey's post-hoc comparisons of group means.

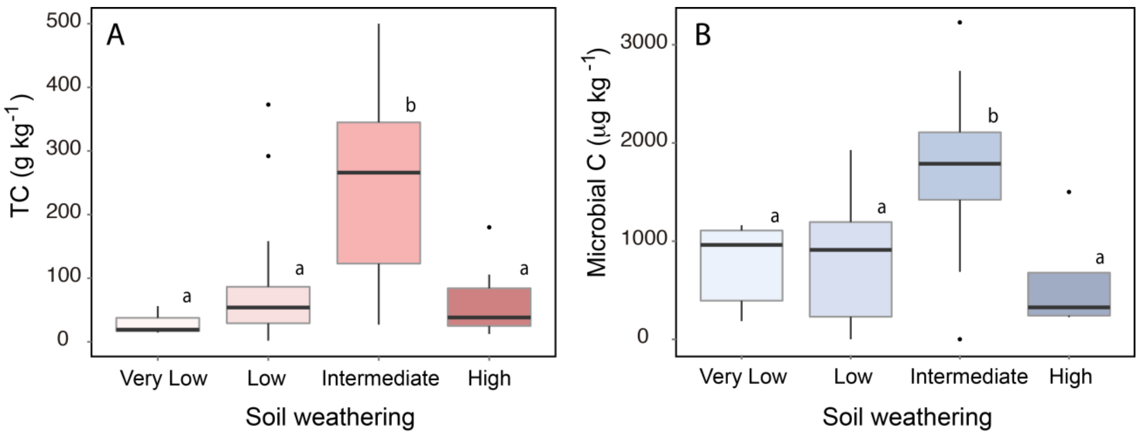
A
TC (g kg-1)
500
400
300
200
100
0
a
a
b
a
Very Low
Low
Intermediate
High
Soil weathering
B
Microbial C (μg kg-1)
3000
2000
1000
0
a
a
b
a
Very Low
Low
Intermediate
High
Soil weathering

Fig S3 Effect of TN across biomes

Partial residual plot of the variability of global Ln phosphatase activity (μmol g^{-1} h^{-1}) explained by Ln TN (g kg^{-1}) (*visreg* R package) for temperate (A), tropical (B) and mediterranean (C) biomes.

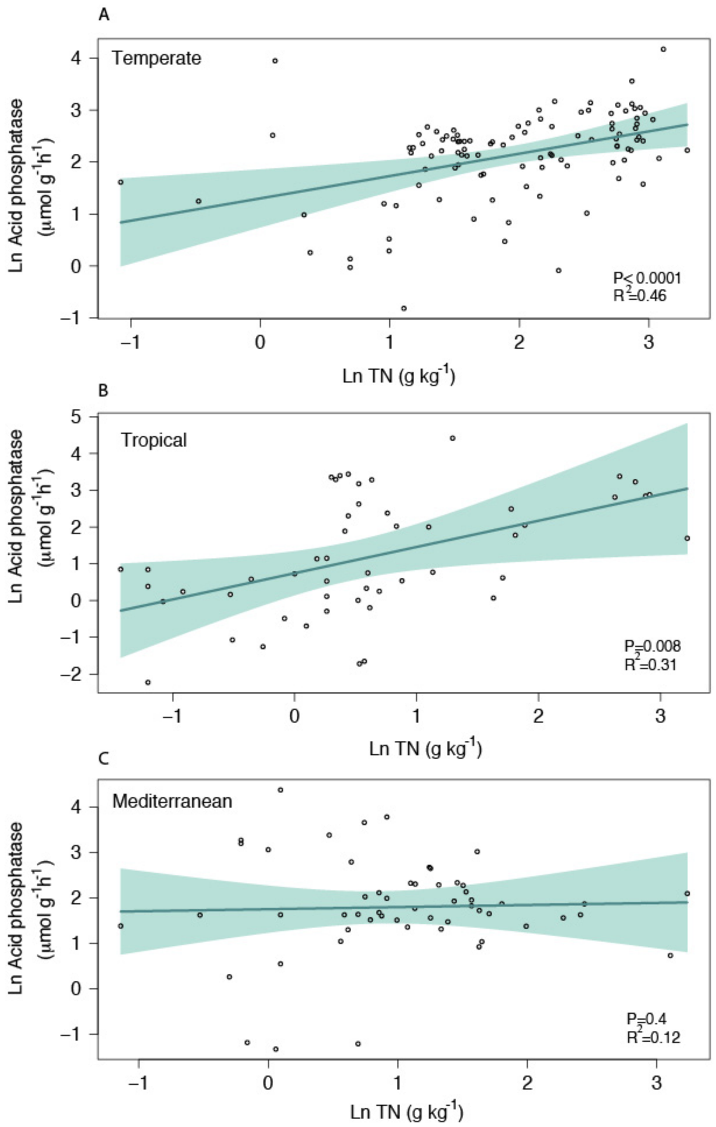

Fig S3 Effect of TN across vegetation type

Scatterplot showing the relationship between Acid Phosphatase and TN. Variables were Ln-transformed. Forest vs. Grassland communities are highlighted. Grassland category includes grasslands and pastures.

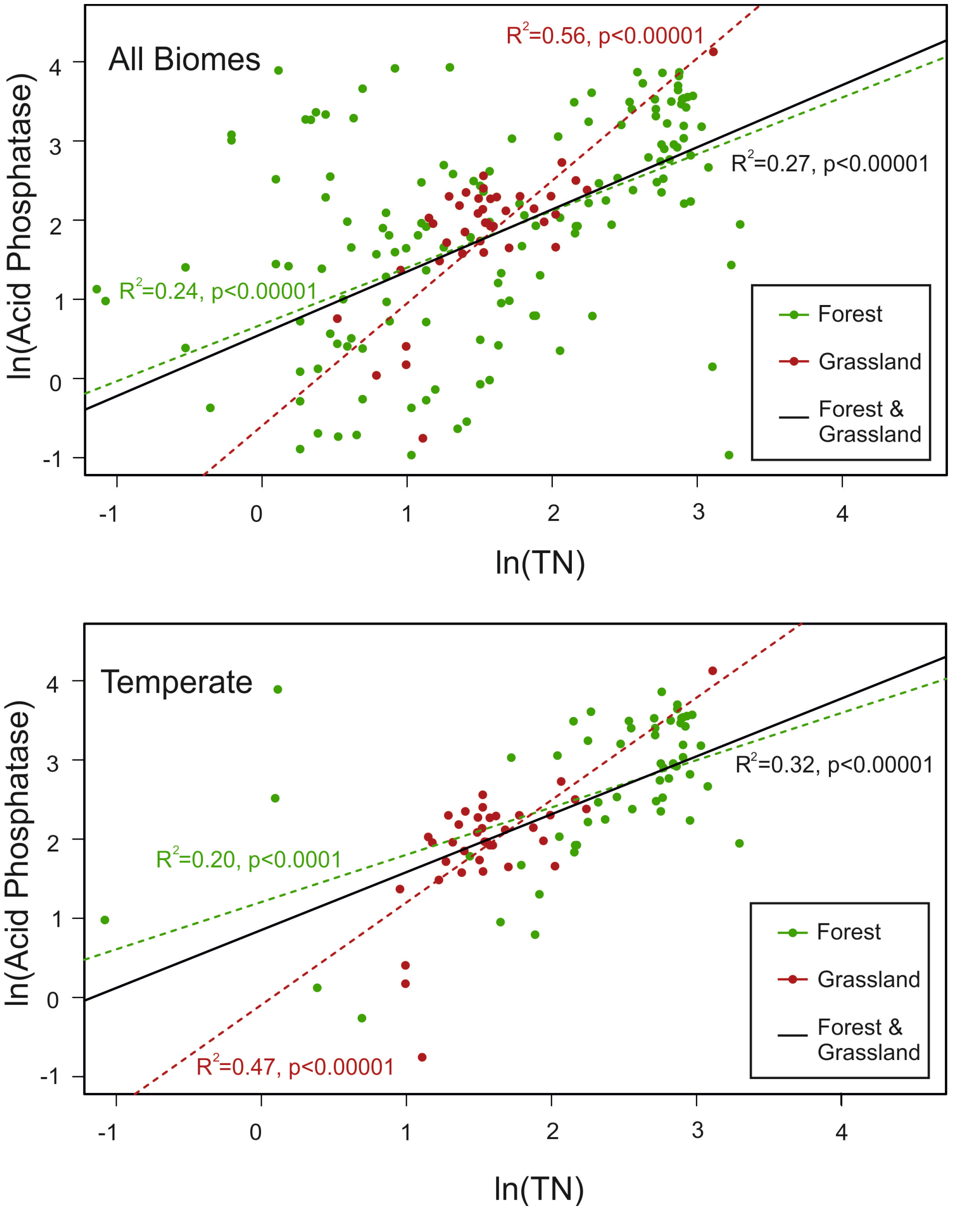

Table S1. Linear model with and without interactions combining TN, MAT, MAP and TN, MAT, MAP, TC and AMP as explanatory variables of acid phosphatase activity. Signif. codes: 0 ‘***’ 0.001 ‘**’ 0.01 ‘*’ 0.05 ‘.’ 0.1 ‘ ’ 1

	Estimate	SE	*t*	*P*	Sig. Code	ßCoeff
Model 1: TN, MAP, MAT with interactions, Multiple R2: 0.27, adjusted R2: 0.26 *F*: 29.04 on 3 and 235 DF, *P*: $5.253e^{-16}$, No. observations in the fit: 239						
Intercept	-52.681	26.39	-1.97	0.0471	*	
Ln TN	0.765	0.091	8.398	4.31E-15	***	0.553
Ln MAP	0.079	0.166	0.477	0.634		0.03
Ln MAT	9.323	4.73	1.97	0.05	.	0.133
Model 2: TN, MAP, MAT with interactions, Multiple R2: 0.30, adjusted R2: 0.28 *F*: 16.4 on 6 and 232 DF, *P*: $9.472e^{-16}$, No. observations in the fit: 239						
Intercept	-754	249	-3.03	0.0027	**	-
Ln TN	0.8	20.9	0.038	0.9695		-
Ln MAP	100.43	35.9	2.8	0.0056	**	-
Ln MAT	133.2	43.9	3.04	0.0027	**	-
Ln TN:Ln MAP	0.053	0.14	0.38	0.71		-
Ln TN:Ln MAP	-0.076	3.75	-0.02	0.98		-
Ln MAT:Ln MAP	-17.7	6.34	-2.8	0.0056	**	-
Model 3 : TN, MAP, MAT, AMP, TC without interactions, Multiple R2: Multiple R2: 0.50, adjusted R2: 0.49 *F*: 21.02 on 5 and 104 DF, *P*: $<1.798e^{-14}$ No. observations in the fit: 110						
Intercept	-52.681	26.39	-1.97	0.0471	**	
Ln TN	0.765	0.091	8.398	4.31E-15	***	0.697
Ln MAP	-0.19	0.329	-0.578	0.564		-0.051
Ln MAT	27.53	7.988	3.446	0.0008	***	0.338
Ln TC	-0.012	0.1454	-0.083	0.933		-0.009
Ln Amp	-1.075	0.659	-1.633	0.1056		-0.161
Model 4 : TN, MAP, MAT, AMP, TC with interactions, Multiple R2: Multiple R2: 0.67, adjusted R2: 0.62 *F*: 12.81 on 15 and 94 DF, *P*: $<2.2e^{-16}$ No. observations in the fit: 110						
Intercept	2888.3565	1199.5565	2.408	0.017999	*	-
Ln TN	133.01929	93.06713	1.429	0.156238		-
Ln MAP	-210.50512	137.94625	-1.526	0.130369		-
Ln MAT	-507.95906	209.42456	-2.425	0.017197	*	-
Ln Amp	-56.13413	71.09077	-0.79	0.431742		-
Ln TC	-523.8962	135.09173	-3.878	0.000195	***	-
Ln TN:Ln MAP	1.04137	0.60578	1.719	0.088896	.	-
Ln TN:Ln MAP	-23.62698	15.47049	-1.527	0.130062		-
Ln MAT:Ln MAP	37.7051	23.88633	1.579	0.117805		-
Ln TN:Ln AMP	-0.09707	0.14196	-0.684	0.495789		-
Ln MAP:Ln AMP	-0.07585	0.36584	-0.207	0.836205		-
Ln MAT:Ln AMP	8.37083	11.9161	0.702	0.484115		-
Ln TN:Ln TC	-1.73328	1.30457	-1.329	0.187189		-
Ln MAP:Ln TC	-1.17335	1.16879	-1.004	0.318003		-
Ln MAT:Ln TC	92.04352	24.20831	3.802	0.000255	***	-
Ln Amp:Ln TC	3.12774	1.03766	3.014	0.003312	**	-

Table S2. Linear model with and without interactions combining TN, MAT, MAP and TN, MAT, MAP, TC and AMP as explanatory variables of acid phosphatase activity for only temperate sites. Signif. codes: 0 ‘***’ 0.001 ‘**’ 0.01 ‘*’ 0.05 ‘.’ 0.1 ‘ ’ 1

	Estimate	SE	*t*	*P*	Sig. Code	ßCoeff
Model 1: TN, MAP, MAT with interactions, Multiple R2: 0.41, adjusted R2: 0.39<br>*F*: 22.98 on 3 and 100 DF, *P*: $2.135e^{-11}$, No. observations in the fit: 104						
Intercept	-131.89	52.032	-2.535	0.0128	*	
Ln TN	0.514	0.1081	4.751	6.78E-06	***	0.416
Ln MAP	0.6506	0.2279	2.855	0.00523	**	0.239
Ln MAT	22.77	9.21	2.47	0.015	*	0.199
Model 2: TN, MAP, MAT with interactions, Multiple R2: 0.46, adjusted R2: 0.42<br>*F*: 13.57 on 6 and 97 DF, *P*: $3.998e^{-11}$ No. observations in the fit: 104						
Intercept	-552	769	-0.717	0.475		-
Ln TN	-90	61.1	-1.47	0.144		-
Ln MAP	78.7	103	0.77	0.446		-
Ln MAT	98	137	0.718	0.475		-
Ln TN:Ln MAP	0.4329	0.34	1.26	0.21		-
Ln TN:Ln MAP	15.5	11	1.4	0.164		-
Ln MAT:Ln MAP	-13.9	18.3	-0.764	0.447		-
Model 3: TN, MAP, MAT, AMP, TC without interactions, Multiple R2: 0.50, adjusted R2: 0.56<br>*F*: 21.22 on 5 and 73 DF, *P*: $<4.853e^{-13}$ No. observations in the fit: 79						
Intercept	-191.5875	79.3329	-2.415	0.0182	*	
Ln TN	0.6765	0.162	4.176	8.11E-05	***	0.517
Ln MAP	0.8366	0.3351	2.496	0.0148	*	0.237
Ln MAT	33.79	13.94	2.424	0.017	*	0.2955
Ln TC	-0.1858	0.1472	-1.262	0.2108		-0.1947
Ln Amp	-1.0891	0.672	-1.621	0.1094		-0.1463
Model 4: TN, MAP, MAT, AMP, TC with interactions, Multiple R2: 0.84, adjusted R2: 0.80<br>*F*: 21.45 on 15 and 63 DF, *P*: $<2.2e^{-16}$, No. observations in the fit: 79						
Intercept	3834.4786	2779.078	1.38	0.17254	***	-
Ln TN	6.3961	137.9777	0.046	0.96317	***	-
Ln MAP	3.3291	315.9972	0.011	0.99163		-
Ln MAT	-666.9843	492.8147	-1.353	0.18076		-
Ln Amp	-120.9173	92.7636	-1.303	0.19715		-
Ln TC	-1150.3699	344.781	-3.337	0.00143	**	-
Ln TN:Ln MAP	0.2948	0.9338	0.316	0.75325		-
Ln TN:Ln MAT	-1.3264	23.8159	-0.056	0.95576		-
Ln MAT:Ln MAP	-2.3051	55.4016	-0.042	0.96694		-
Ln TN:Ln AMP	-0.4588	0.1509	-3.041	0.00343	**	-
Ln MAP:Ln AMP	0.6284	0.809	0.777	0.4402		-
Ln MAT:Ln AMP	20.9023	15.639	1.337	0.18618		-
Ln TN:Ln TC	0.6802	1.6586	0.41	0.68315		-
Ln MAP:Ln TC	2.2726	3.3639	0.676	0.50178		-
Ln MAT:Ln TC	200.6906	62.6777	3.202	0.00214	**	-
Ln Amp:Ln TC	-0.2184	1.4342	-0.152	0.87947		-

Table S3. Linear model with interactions and including TN, MAT, MAP and pH as explanatory variables of alkaline phosphatase activity. Signif. codes: 0 ‘***’ 0.001 ‘**’ 0.01 ‘*’ 0.05 ‘.’ 0.1 ‘ ’ 1

	Estimate	SE	*t*	*P*	Sig. Code
Model alkaline: TN, MAP, MAT and pH with interactions, Multiple R2: 0.56, adjusted R2: 0.42 *F*: 4.246 on 15 and 51 DF, *P*: 5.099e^{-05}, No. observations in the fit: 67					
Intercept	21061.3	5784.8	3.64	0.000635	***
Ln TN	-8225.5	5729.4	-1.44	0.157205	
Ln MAP	-2817.6	784.7	-3.59	0.000741	***
Ln MAT	-3717	1022.5	-3.64	0.000645	***
Ln pH	-10376.5	2839.8	-3.65	0.00061	***
Ln TN:Ln MAP	1283.4	801	1.60	0.115289	
Ln TN:Ln MAT	1437.5	1012.1	1.42	0.161562	
Ln MAT:Ln MAP	497.1	138.6	3.59	0.00075	***
Ln TN:Ln pH	5271.1	2966.6	1.78	0.081567	.
Ln MAP:Ln pH	1391.9	391.6	3.55	0.000828	***
Ln MAT:Ln pH	1831.6	501.9	3.65	0.000619	***
Ln TN:Ln MAP:Ln MAT	-224.5	141.4	-1.59	0.118556	
Ln TN:Ln MAP:Ln pH	-810.3	422.8	-1.92	0.060913	.
Ln TN:Ln MAT:Ln pH	-922.4	523	-1.76	0.084282	.
Ln MAP:Ln MAT:Ln pH	-245.5	69.1	-3.55	0.000838	***
Ln TN: Ln MAP:Ln MAT:Ln pH	141.9	74.6	1.90	0.062919	.

Database literature

1. Acosta-Martínez, V., Cruz, L., Sotomayor-Ramírez, D. & Pérez-Alegría, L. Enzyme activities as affected by soil properties and land use in a tropical watershed. *Appl. Soil Ecol.* **35,** 35–45 (2007).
2. Adamczyk, B., Adamczyk, S., Kukkola, M., Tamminen, P. & Smolander, A. Logging residue harvest may decrease enzymatic activity of boreal forest soils. *Soil Biol. Biochem.* **82,** 74–80 (2015).
3. Adamczyk, B., Kilpeläinen, P., Kitunen, V. & Smolander, A. Potential activities of enzymes involved in N, C, P and S cycling in boreal forest soil under different tree species. *Pedobiologia (Jena).* **57,** 97–102 (2014).
4. Ajwa, H. A., Dell, C. J. & Rice, C. W. Changes in enzyme activities and microbial biomass of tallgrass prairie soil as related to burning and nitrogen fertilization. *Soil Biol. Biochem.* **31,** 769–777 (1999).
5. Allison, S. D., Nielsen, C. & Hughes, R. F. Elevated enzyme activities in soils under the invasive nitrogen-fixing tree Falcataria moluccana. *Soil Biol. Biochem.* **38,** 1537–1544 (2006).
6. An, S., Cheng, Y., Huang, Y. & Liu, D. Effects of revegetation on soil microbial biomass, enzyme activities, and nutrient cycling on the Loess Plateau in China. *Restor. Ecol.* **21,** 600–607 (2013).
7. Antibus, R. K. Effects of Liming a Red Pine Forest Floor on Numbers and Mycorrhizal and Soil Acid Phosphatase Activities. **24,** (1992).
8. Antunes, S. C., Curado, N., Castro, B. B. & Gonçalves, F. Short-term recovery of soil functional parameters and edaphic macro-arthropod community after a forest fire. *J. Soils Sediments* **9,** 267–278 (2009).
9. Baena, C. W. *et al.* Thinning and recovery effects on soil properties in two sites of a Mediterranean forest, in Cuenca Mountain (South-eastern of Spain). *For. Ecol. Manage.* **308,** 223–230 (2013).
10. Bai, G., Bao, Y., Du, G. & Qi, Y. Arbuscular mycorrhizal fungi associated with vegetation and soil parameters under rest grazing management in a desert steppe ecosystem. *Mycorrhiza* **23,** 289–301 (2013).
11. Bai, C., He, X., Tang, H., Shan, B. & Zhao, L. Spatial distribution of arbuscular mycorrhizal fungi, glomalin and soil enzymes under the canopy of Astragalus adsurgens Pall. in the Mu Us sandland, China. *Soil Biol. Biochem.* **41,** 941–947 (2009).
12. Baldrian, P. *et al.* Responses of the extracellular enzyme activities in hardwood forest to soil temperature and seasonality and the potential effects of climate change. *Soil Biol. Biochem.* **56,** 60–68 (2013).
13. Baldrian, P. *et al.* Enzyme activities and microbial biomass in topsoil layer during spontaneous succession in spoil heaps after brown coal mining. *Soil Biol. Biochem.* **40,** 2107–2115 (2008).
14. Baldrian, P., Merhautová, V., Cajthaml, T., Petránková, M. & Šnajdr, J. Small-scale distribution of extracellular enzymes, fungal, and bacterial biomass in Quercus petraea forest topsoil. *Biol. Fertil. Soils* **46,** 717–726 (2010).
15. Bastida, F. *et al.* Application of fresh and composted organic wastes modifies structure, size and activity of soil microbial community under semiarid climate. *Appl. Soil Ecol.* **40,** 318–329 (2008).
16. Bastida, F., Moreno, J. L., Hernández, T. & García, C. The long-term effects of the management of a forest soil on its carbon content, microbial biomass and activity under a semi-arid climate. *Appl. Soil Ecol.* **37,** 53–62 (2007).
17. Bastida, F., Luis Moreno, J., Teresa Hernández & García, C. Microbiological degradation index of soils in a semiarid climate. *Soil Biol. Biochem.* **38,** 3463–3473 (2006).
18. Baum, C. & Hrynkiewicz, K. Clonal and seasonal shifts in communities of saprotrophic microfungi and soil enzyme activities in the mycorrhizosphere of Salix spp. *J. Plant Nutr. Soil Sci.* **169,** 481–487 (2006).
19. Bielińska, E. J., Mocek-Płóciniak, A. & Kaczmarek, Z. Eco-Chemical forest soil indices from a forest fire. *Polish J. Environ. Stud.* **17,** 665–671 (2008).
20. Bilgo, A. *et al.* Response of native soil microbial functions to the controlled mycorrhization of an exotic tree legume, Acacia holosericea in a Sahelian ecosystem. *Mycorrhiza* **22,** 175–187 (2012).
21. Blanes, M. C., Viñegla, B., Salido, M. T. & Carreira, J. A. Coupled soil-availability and tree-limitation nutritional shifts induced by N deposition: insights from N to P relationships in Abies pinsapo forests. *Plant Soil* **366,** 67–81 (2013).
22. Blank, R. R., Chambers, J. C. & Zamudio, D. Restoring riparian corridors with fire: Effects on soil and vegetation. *J. Range Manag.* 388–396 (2003).
23. Boerner, R. E. J. & Brinkman, J. A. Fire frequency and soil enzyme activity in southern Ohio oak-hickory forests. *Appl. Soil Ecol.* **23,** 137–146 (2003).
24. Boerner, R. E. J., Brinkman, J. A. & Smith, A. Seasonal variations in enzyme activity and organic carbon in soil of a burned and unburned hardwood forest. *Soil Biol. Biochem.* **37,** 1419–1426 (2005).
25. Boerner, R. E. J., Coates, A. T., Yaussy, D. A. & Waldrop, T. A. Assessing ecosystem restoration alternatives in eastern deciduous forests: the view from belowground. *Restor. Ecol.* **16,** 425–434 (2008).
26. Boerner, R. E. J., Decker, K. L. M. & Sutherland, E. K. Prescribed burning effects on soil enzyme activity in a southern Ohio hardwood forest: a landscape-scale analysis. *Soil Biol. Biochem.* **32,** 899–908 (2000).
27. Böhme, L., Langer, U. & Böhme, F. Microbial biomass, enzyme activities and microbial community structure in two European long-term field experiments. *Agric. Ecosyst. Environ.* **109,** 141–152 (2005).
28. Caravaca, F., Alguacil, M. M., Figueroa, D., Barea, J. M. & Roldán, A. Re-establishment of Retama sphaerocarpa as a target species for reclamation of soil physical and biological properties in a semi-arid Mediterranean area. *For.*

Ecol. Manage. **182,** 49–58 (2003).
29. Carline, K. A., Jones, H. E. & Bardgett, R. D. Large herbivores affect the stoichiometry of nutrients in a regenerating woodland ecosystem. *Oikos* **110,** 453–460 (2005).
30. Carreira, J. A. & Lajtha, K. Factors affecting phosphate sorption along a Mediterranean, dolomitic soil and vegetation chronosequence. *Eur. J. Soil Sci.* **48,** 139–149 (1997).
31. Carreira, J. A., Lajtha, K. & Niell, F. X. Phosphorus transformations along a soil/vegetation series of fire-prone, dolomitic, semi-arid shrublands of southern spain soil p and mediterranean shrubland dynamic. *Biogeochemistry* **39,** 87–120 (1997).
32. Chacon, N., Flores, S. & Gonzalez, A. Implications of iron solubilization on soil phosphorus release in seasonally flooded forests of the lower Orinoco River, Venezuela. *Soil Biol. Biochem.* **38,** 1494–1499 (2006).
33. Chang, E.-H., Chen, C.-T., Chen, T.-H. & Chiu, C.-Y. Soil microbial communities and activities in sand dunes of subtropical coastal forests. *Appl. soil Ecol.* **49,** 256–262 (2011).
34. Chen, C. R., Condron, L. M., Davis, M. R. & Sherlock, R. R. Effects of afforestation on phosphorus dynamics and biological properties in a New Zealand grassland soil. *Plant Soil* **220,** 151–163 (2000).
35. Chen, H. Phosphatase activity and P fractions in soils of an 18-year-old Chinese fir (Cunninghamia lanceolata) plantation. *For. Ecol. Manage.* **178,** 301–310 (2003).
36. Cheng, F. *et al.* Soil Microbial Biomass, Basal Respiration and Enzyme Activity of Main Forest Types in the Qinling Mountains. *PLoS One* **8,** (2013).
37. Clarholm, M. Microbial Biomass-P, Labile-P, and Acid-Phosphatase-Activity in the Humus Layer of a Spruce Forest, After Repeated Additions of Fertilizers. *Biol. Fertil. Soils* **16,** 287–292 (1993).
38. Closa, I. & Goicoechea, N. Seasonal dynamics of the physicochemical and biological properties of soils in naturally regenerating, unmanaged and clear-cut beech stands in northern Spain. *Eur. J. Soil Biol.* **46,** 190–199 (2010).
39. Colvan, S. R., Syers, J. K. & O'Donnell, a G. O. Effect of long-term fertiliser use on acid and alkaline phosphomonoesterase and phosphodiesterase activities in managed grassland. *Biol. Fertil. Soils* **34,** 258–263 (2001).
40. Costa, D., Freitas, H. & Sousa, J. P. Influence of seasons and land-use practices on soil microbial activity and metabolic diversity in the 'Montado ecosystem'. *Eur. J. Soil Biol.* **59,** 22–30 (2013).
41. de Carvalho Mendes, I., Fernandes, M. F., Chaer, G. M. & dos Reis Junior, F. B. Biological functioning of Brazilian Cerrado soils under different vegetation types. *Plant Soil* **359,** 183–195 (2012).
42. Defrieri, R. L., Effron, D., Jimenez, M. P. & Prause, J. Enzimas Fosfatasa Ácida Y Proteasas En Un Suelo De Bosque Influence of Tree Species on the Activity of Acid Phosphatase and Protease. **26,** 177–182 (2008).
43. Alguacil, M. del M., Lozano, Z., Campoy, M. J. & Roldán, A. Phosphorus fertilisation management modifies the biodiversity of AM fungi in a tropical savanna forage system. *Soil Biol. Biochem.* **42,** 1114–1122 (2010).
44. Dillard, S. L. *et al.* Effects of nitrogen fertilization on soil nutrient concentration and phosphatase activity and forage nutrient uptake from a grazed pasture system. *J. Environ. Manage.* **154,** 208–215 (2015).
45. Dilly, O. & Nannipieri, P. Response of ATP content, respiration rate and enzyme activities in an arable and a forest soil to nutrient additions. *Biol. Fertil. Soils* **34,** 64–72 (2001).
46. Dinesh, R., Ghoshal Chaudhuri, S. & Sheeja, T. E. Soil biochemical and microbial indices in wet tropical forests: Effects of deforestation and cultivation. *J. Plant Nutr. Soil Sci.* **167,** 24–32 (2004).
47. Drissner, D., Blum, H., Tscherko, D. & Kandeler, E. Nine years of enriched CO2 changes the function and structural diversity of soil microorganisms in a grassland. *Eur. J. Soil Sci.* **58,** 260–269 (2007).
48. Effron, D. N., Jiménez, M. P., Defrieri, R. L. & Prause, J. Relación de la actividad de fosfatasa ácida con especies forestales dominantes y con algunas propiedades del suelo de un bosque argentino. *Inf. tecnológica* **17,** 3–7 (2006).
49. Figueira da Silva, C. *et al.* Carbono orgânico total, biomassa microbiana e atividade enzimática do solo de áreas agrícolas, florestais e pastagem no médio vale do paraíba do sul (RJ). *Rev. Bras. Ciência do Solo* **36,** (2012).
50. Fioretto, A., Papa, S., Pellegrino, A. & Ferrigno, A. Microbial activities in soils of a Mediterranean ecosystem in different successional stages. *Soil Biol. Biochem.* **41,** 2061–2068 (2009).
51. Fontúrbel, M. T. *et al.* Effects of an experimental fire and post-fire stabilization treatments on soil microbial communities. *Geoderma* **191,** 51–60 (2012).
52. Garcia-Franco, N., Wiesmeier, M., Goberna, M., Martínez-Mena, M. & Albaladejo, J. Carbon dynamics after afforestation of semiarid shrublands: Implications of site preparation techniques. *For. Ecol. Manage.* **319,** 107–115 (2014).
53. García-Morote, F. A. *et al.* Effects of woodland maturity, vegetation cover and season on enzymatic and microbial activity in thermophilic Spanish juniper woodlands (Juniperus thurifera L.) of southern Spain. *Eur. J. Soil Sci.* **63,** 579–591 (2012).
54. Geng, Y., Dighton, J. & Gray, D. The effects of thinning and soil disturbance on enzyme activities under pitch pine soil in New Jersey Pinelands. *Appl. soil Ecol.* **62,** 1–7 (2012).
55. Giai, C. & Boerner, R. E. J. Effects of ecological restoration on microbial activity, microbial functional diversity, and soil organic matter in mixed-oak forests of southern Ohio, USA. *Appl. Soil Ecol.* **35,** 281–290 (2007).
56. Gispert, M., Emran, M., Pardini, G., Doni, S. & Ceccanti, B. The impact of land management and abandonment on soil enzymatic activity, glomalin content and aggregate stability. *Geoderma* **202-203,** 51–61 (2013).
57. Goberna, M., Sánchez, J., Pascual, J. A. & García, C. Pinus halepensis Mill. plantations did not restore organic

carbon, microbial biomass and activity levels in a semi-arid Mediterranean soil. *Appl. Soil Ecol.* **36,** 107–115 (2007).

58. Gonnety, J. T. *et al.* Effect of land-use types on soil enzymatic activities and chemical properties in semi-deciduous forest areas of Central-West Cote d'Ivoire. *Biotechnol. Agron. Soc. Environ.* **16,** 478–485 (2012).
59. Grego, S. *et al.* Mediterranean natural forest living at elevated carbon dioxide: soil biological properties and plant biomass growth. *Soil Use Manag.* **17,** 195–202 (2001).
60. Grierson, P. F. & Adams, M. A. Plant species affect acid phosphatase, ergosterol and microbial P in a Jarrah (Eucalyptus marginata Donn ex Sm.) forest in south-western Australia. *Soil Biol. Biochem.* **32,** 1817–1827 (2000).
61. Griffiths, R. P. & Filan, T. Effects of Bracken Fern Invasions on Harvested Site Soils in Pacific Northwest (USA) Coniferous Forests. *Northwest Sci.* **81,** 191–198 (2007).
62. Groffman, P. M. & Fisk, M. C. Phosphate additions have no effect on microbial biomass and activity in a northern hardwood forest. *Soil Biol. Biochem.* **43,** 2441–2449 (2011).
63. Guenet, B. *et al.* The impact of long-term CO2 enrichment and moisture levels on soil microbial community structure and enzyme activities. *Geoderma* **170,** 331–336 (2012).
64. Guo, P. *et al.* Mixed inorganic and organic nitrogen addition enhanced extracellular enzymatic activities in a subtropical forest soil in east China. *Water, Air, Soil Pollut.* **216,** 229–237 (2011).
65. Guo, Y. J. & Han, J. G. Soil biochemical properties and arbuscular mycorrhizal fungi as affected by afforestation of rangelands in northern China. *J. Arid Environ.* **72,** 1690–1697 (2008).
66. Hamman, S. T., Burke, I. C. & Knapp, E. E. Soil nutrients and microbial activity after early and late season prescribed burns in a Sierra Nevada mixed conifer forest. *For. Ecol. Manage.* **256,** 367–374 (2008).
67. Han, J., Jung, J., Hyun, S., Park, H. & Park, W. Effects of nutritional input and diesel contamination on soil enzyme activities and microbial communities in antarctic soils. *J. Microbiol.* **50,** 916–924 (2012).
68. Hedo, J., Lucas-Borja, M. E., Wic-Baena, C., Andrés-Abellán, M. & de las Heras, J. Experimental site and season over-control the effect of Pinus halepensis in microbiological properties of soils under semiarid and dry conditions. *J. Arid Environ.* **116,** 44–52 (2015).
69. Hinojosa, M. B. *et al.* Effects of drought on soil phosphorus availability and fluxes in a burned Mediterranean shrubland. *Geoderma* **191,** 61–69 (2012).
70. Hou, E., Chen, C., Wen, D. & Liu, X. Phosphatase activity in relation to key litter and soil properties in mature subtropical forests in China. *Sci. Total Environ.* **515-516,** 83–91 (2015).
71. Huang, W. J. *et al.* Responses of Soil Acid Phosphomonoesterase Activity to Simulated Nitrogen Deposition in Three Forests of Subtropical China. *Pedosphere* **22,** 698–706 (2012).
72. Huang, W. *et al.* Increasing phosphorus limitation along three successional forests in southern China. *Plant Soil* **364,** 181–191 (2013).
73. Huang, W. *et al.* Short-term effects of prescribed burning on phosphorus availability in a suburban native forest of subtropical Australia. *J. Soils Sediments* **13,** 869–876 (2013).
74. Huang, W., Liu, J., Zhou, G., Zhang, D. & Deng, Q. Effects of precipitation on soil acid phosphatase activity in three successional forests in southern China. *Biogeosciences* **8,** 1901–1910 (2011).
75. Imai, N., Kitayama, K. & Titin, J. Effects of logging on phosphorus pools in a tropical rainforest of Borneo. *J. Trop. For. Sci.* **24,** 5–17 (2012).
76. Izaguirre-Mayoral, M. L., Flores, S. & Carballo, O. Determination of acid phosphatase and dehydrogenase activities in the rhizosphere of nodulated legume species native to two contrasting savanna sites in Venezuela. *Biol. Fertil. Soils* **35,** 470–472 (2002).
77. Jones, A. G. & Davidson, N. J. Altered N, P and C dynamics with absence of fire in Eucalyptus forests affected by premature decline. *Austral Ecol.* **39,** 587–599 (2014).
78. Kandeler, E. *et al.* Response of soil microbial biomass and enzyme activities to the transient elevation of carbon dioxide in a semi-arid grassland. *Soil Biol. Biochem.* **38,** 2448–2460 (2006).
79. Kitayama, K. The activities of soil and root acid phosphatase in the nine tropical rain forests that differ in phosphorus availability on Mount Kinabalu, Borneo. *Plant Soil* **367,** 215–224 (2013).
80. Klose, S., Wernecke, K. D. & Makeschin, F. Microbial activities in forest soils exposed to chronic depositions from a lignite power plant. *Soil Biol. Biochem.* **36,** 1913–1923 (2004).
81. Koné, A. W. *et al.* Can the shrub Chromolaena odorata (Asteraceae) be considered as improving soil biology and plant nutrient availability? *Agrofor. Syst.* **85,** 233–245 (2012).
82. Kunito, T., Tobitani, T., Moro, H. & Toda, H. Phosphorus limitation in microorganisms leads to high phosphomonoesterase activity in acid forest soils. *Pedobiologia (Jena).* **55,** 263–270 (2012).
83. Kuperman, R. G. & Carreiro, M. M. Soil heavy metal concentrations, microbial biomass and enzyme activities in a contaminated grassland ecosystem. *Soil Biol. Biochem.* **29,** 179–190 (1997).
84. Lebrun, J. D. *et al.* Assessing impacts of copper on soil enzyme activities in regard to their natural spatiotemporal variation under long-term different land uses. *Soil Biol. Biochem.* **49,** 150–156 (2012).
85. Lee, Y. K. *et al.* Effect of Acacia plantations on net photosynthesis, tree species composition, soil enzyme activities, and microclimate on Mt. Makiling. *Photosynthetica* **44,** 299–308 (2006).
86. Leirós, M. C., Trasar-Cepeda, C., Seoane, S. & Gil-Sotres, F. Biochemical properties of acid soils under climax vegetation (Atlantic oakwood) in an area of the European temperate–humid zone (Galicia, NW Spain): general

parameters. *Soil Biol. Biochem.* **32,** 733–745 (2000).
87. Li, S. *et al.* Effects of different types of N deposition on the fungal decomposition activities of temperate forest soils. *Sci. Total Environ.* **497-498,** 91–96 (2014).
88. Li, W., Zhang, C., Jiang, H., Xin, G. & Yang, Z. Changes in soil microbial community associated with invasion of the exotic weed, Mikania micrantha HBK. *Plant Soil* **281,** 309–324 (2006).
89. Li, X. G. *et al.* Dynamics of soil properties and organic carbon pool in topsoil of zokor-made mounds at an alpine site of the Qinghai-Tibetan plateau. *Biol. Fertil. Soils* **45,** 865–872 (2009).
90. Li, X. & Sarah, P. Enzyme activities along a climatic transect in the Judean Desert. *Catena* **53,** 349–363 (2003).
91. Lillo, A., Ramírez, H., Reyes, F., Ojeda, N. & Alvear, M. Actividad biológica del suelo de bosque templado en un transecto altitudinal, Parque Nacional Conguillío (38° S), Chile. *Bosque (Valdivia)* **32,** 46–56 (2011).
92. Lü, Y. *et al.* Responses of soil microbial biomass and enzymatic activities to different forms of organic nitrogen deposition in the subtropical forests in East China. *Ecol. Res.* **28,** 447–457 (2013).
93. Lucas-Borja, M. E. *et al.* The Effects of Human Trampling on the Microbiological Properties of Soil and Vegetation in Mediterranean Mountain Areas. *L. Degrad. Dev.* **22,** 383–394 (2011).
94. Lucas-Borja, M. E. *et al.* Influence of forest cover and herbaceous vegetation on the microbiological and biochemical properties of soil under Mediterranean humid climate. *Eur. J. Soil Biol.* **46,** 273–279 (2010).
95. Lucas-Borja, M. E., Candel Pérez, D., López Serrano, F. R., Andrés, M. & Bastida, F. Altitude-related factors but not Pinus community exert a dominant role over chemical and microbiological properties of a Mediterranean humid soil. *Eur. J. Soil Sci.* **63,** 541–549 (2012).
96. Lucas-Borja, M. E. *et al.* Microbial activity in soils under fast-growing Paulownia (Paulownia elongata x fortunei) plantations in Mediterranean areas. *Appl. Soil Ecol.* **51,** 42–51 (2011).
97. Lv, Y. *et al.* Effects of nitrogen addition on litter decomposition, soil microbial biomass, and enzyme activities between leguminous and non-leguminous forests. *Ecol. Res.* **28,** 793–800 (2013).
98. Margesin, R., Minerbi, S. & Schinner, F. Long-Term Monitoring of Soil Microbiological Activities in Two Forest Sites in South Tyrol in the Italian Alps. *Microbes Environ.* **29,** 277–285 (2014).
99. Marinari, S. & Antisari, L. V. Effect of lithological substrate on microbial biomass and enzyme activity in brown soil profiles in the northern Apennines (Italy). *Pedobiologia (Jena).* **53,** 313–320 (2010).
100. Matinizadeh, M., Korori, S. A. A., Teimouri, M. & Praznik, W. Enzyme activities in undisturbed and disturbed forest soils under oak (Quercus brantii var. persica) as affected by soil depth and seasonal variation. *Asian J. Plant Sci.* (2008).
101. Meason, D. F., Idol, T. W., Friday, J. B. & Scowcroft, P. G. Effects of fertilisation on phosphorus pools in the volcanic soil of a managed tropical forest. *For. Ecol. Manage.* **258,** 2199–2206 (2009).
102. Menge, D. N. L. & Field, C. B. Simulated global changes alter phosphorus demand in annual grassland. *Glob. Chang. Biol.* **13,** 2582–2591 (2007).
103. Mijangos, I. *et al.* Effects of liming on soil properties and plant performance of temperate mountainous grasslands. *J. Environ. Manage.* **91,** 2066–2074 (2010).
104. Mora, P., Miambi, E., Jiménez, J. J., Decaëns, T. & Rouland, C. Functional complement of biogenic structures produced by earthworms, termites and ants in the neotropical savannas. *Soil Biol. Biochem.* **37,** 1043–1048 (2005).
105. Moreno, J. L., Garcia, C. & Hernandez, T. Toxic effect of cadmium and nickel on soil enzymes and the influence of adding sewage sludge. *Eur. J. Soil Sci.* **54,** 377–386 (2003).
106. Moscatelli, M. C., Lagomarsino, A., De Angelis, P. & Grego, S. Seasonality of soil biological properties in a poplar plantation growing under elevated atmospheric CO2. *Appl. Soil Ecol.* **30,** 162–173 (2005).
107. Muscolo, A., Sidari, M. & Mercurio, R. Influence of gap size on organic matter decomposition, microbial biomass and nutrient cycle in Calabrian pine (Pinus laricio, Poiret) stands. *For. Ecol. Manage.* **242,** 412–418 (2007).
108. Naples, B. K. & Fisk, M. C. Belowground insights into nutrient limitation in northern hardwood forests. *Biogeochemistry* **97,** 109–121 (2010).
109. Neitzke, M. Bodenbiologische Parameter entlang von Transekten in calcareous grasslands. (1999).
110. Ndour, N. Y. B. *et al.* Characteristics of microbial habitats in a tropical soil subject to different fallow management. *Appl. soil Ecol.* **38,** 51–61 (2008).
111. Nosrati, K., Govers, G. & Smolders, E. Dissolved organic carbon concentrations and fluxes correlate with land use and catchment characteristics in a semi-arid drainage basin of Iran. *Catena* **95,** 177–183 (2012).
112. Olander, L. P. & Vitousek, P. M. Regulation of soil phosphatase and chitinase activity by N and P availability. *Biogeochemistry* **49,** 175–190 (2000).
113. Orczewska, A., Piotrowska, A. & Lemanowicz, J. Soil acid phosphomonoesterase activity and phosphorus forms in ancient and post-agricultural black alder [Alnus glutinosa (L.) Gaertn.] woodlands. *Acta Soc. Bot. Pol.* **81,** 81–86 (2012).
114. Pan, C., Liu, C., Zhao, H. & Wang, Y. Changes of soil physico-chemical properties and enzyme activities in relation to grassland salinization. *Eur. J. Soil Biol.* **55,** 13–19 (2013).
115. Panwar, J., Saini, V. K., Tarafdar, J. C., Kumar, P. & Kathju, S. Changes in labile P status under different cropping systems in an arid environment. *J. Arid Environ.* **61,** 137–145 (2005).
116. Paz-Ferreiro, J., Trasar-Cepeda, C., Leirós, M. C., Seoane, S. & Gil-Sotres, F. Biochemical properties in managed

grassland soils in a temperate humid zone: Modifications of soil quality as a consequence of intensive grassland use. *Biol. Fertil. Soils* **45,** 711–722 (2009).

117. Pourreza, M., Hosseini, S. M., Safari Sinegani, A. A., Matinizadeh, M. & Dick, W. A. Soil microbial activity in response to fire severity in Zagros oak (Quercus brantii Lindl.) forests, Iran, after one year. *Geoderma* **213,** 95–102 (2014).
118. Prieto, L. H., Bertiller, M. B., Carrera, A. L. & Olivera, N. L. Soil enzyme and microbial activities in a grazing ecosystem of Patagonian Monte, Argentina. *Geoderma* **162,** 281–287 (2011).
119. Raiesi, F. & Beheshti, A. Soil C turnover, microbial biomass and respiration, and enzymatic activities following rangeland conversion to wheat-alfalfa cropping in a semi-arid climate. *Environ. Earth Sci.* **72,** 5073–5088 (2014).
120. Raiesi, F. & Beheshti, A. Soil specific enzyme activity shows more clearly soil responses to paddy rice cultivation than absolute enzyme activity in primary forests of northwest Iran. *Appl. Soil Ecol.* **75,** 63–70 (2014).
121. Raiesi, F. & Riahi, M. The influence of grazing exclosure on soil C stocks and dynamics, and ecological indicators in upland arid and semi-arid rangelands. *Ecol. Indic.* **41,** 145–154 (2014).
122. Redel, Y., Rubio, R., Godoy, R. & Borie, F. Phosphorus fractions and phosphatase activity in an Andisol under different forest ecosystems. *Geoderma* **145,** 216–221 (2008).
123. Renella, G., Landi, L., Valori, F. & Nannipieri, P. Microbial and hydrolase activity after release of low molecular weight organic compounds by a model root surface in a clayey and a sandy soil. *Appl. Soil Ecol.* **36,** 124–129 (2007).
124. Reyes, F., Lillo, A., Ojeda, N., Reyes, M. & Alvear, M. Efecto de la exposición y la toposecuencia sobre actividades biológicas del suelo en bosque relicto del centro-sur de Chile. *Bosque (Valdivia)* **32,** 255–265 (2011).
125. Rivas, Y. *et al.* Actividad biológica del suelo en dos bosques de Nothofagus del centro sur de Chile. *Gayana. Botánica* **64,** 81–92 (2007).
126. Roose-Amsaleg, C., Mora, P. & Harry, M. Physical, chemical and phosphatase activities characteristics in soil-feeding termite nests and tropical rainforest soils. *Soil Biol. Biochem.* **37,** 1910–1917 (2005).
127. Ross, D. J., Speir, T. W., Tate, K. R. & Feltham, C. W. Burning in a New Zealand snow-tussock grassland: effects on soil microbial biomass and nitrogen and phosphorus availability. *N. Z. J. Ecol.* 63–71 (1997).
128. Rui, Y. *et al.* Warming and grazing increase mineralization of organic P in an alpine meadow ecosystem of Qinghai-Tibet Plateau, China. *Plant Soil* **357,** 73–87 (2012).
129. Saa, A., Trasar-Cepeda, M. C., Gil-Sotres, F. & Carballas, T. Changes in soil phosphorus and acid phosphatase activity immediately following forest fires. *Soil Biol. Biochem.* **25,** 1223–1230 (1993).
130. Salam, A. K. *et al.* Activities of soil enzymes in different land-use systems in middle terrace areas of Lampung Province, South Sumatra, Indonesia. *Soil Sci. plant Nutr.* **45,** 89–99 (1999).
131. Salam, A. K., Katayama, A. & Kimura, M. Activities of some soil enzymes in different land use systems after deforestation in hilly areas of West Lampung, South Sumatra, Indonesia. *Soil Sci. Plant Nutr.* **44,** 93–103 (1998).
132. Sandoval-Pérez, A. L., Gavito, M. E., García-Oliva, F. & Jaramillo, V. J. Carbon, nitrogen, phosphorus and enzymatic activity under different land uses in a tropical, dry ecosystem. *Soil Use Manag.* **25,** 419–426 (2009).
133. Sardans, J. & Peñuelas, J. Drought decreases soil enzyme activity in a Mediterranean Quercus ilex L. forest. *Soil Biol. Biochem.* **37,** 455–461 (2005).
134. Sardans, J., Peñuelas, J. & Estiarte, M. Warming and drought alter soil phosphatase activity and soil P availability in a Mediterranean shrubland. *Plant Soil* **289,** 227–238 (2006).
135. Sardans, J., Peñuelas, J. & Ogaya, R. Experimental drought reduced acid and alkaline phosphatase activity and increased organic extractable P in soil in a Quercus ilex Mediterranean forest. *Eur. J. Soil Biol.* **44,** 509–520 (2008).
136. Satti, P., Mazzarino, M. J., Roselli, L. & Crego, P. Factors affecting soil P dynamics in temperate volcanic soils of southern Argentina. *Geoderma* **139,** 229–240 (2007).
137. Saviozzi, A., Levi-Minzi, R., Cardelli, R. & Riffaldi, R. A comparison of soil quality in adjacent cultivated, forest and native grassland soils. *Plant Soil* **233,** 251–259 (2001).
138. Schneider, K., Turrion, M. B., Grierson, P. F. & Gallardo, J. F. Phosphatase activity, microbial phosphorus, and fine root growth in forest soils in the Sierra de Gata, western central Spain. *Biol. Fertil. Soils* **34,** 151–155 (2001).
139. Scott, J. T. & Condron, L. M. Dynamics and availability of phosphorus in the rhizosphere of a temperate silvopastoral system. *Biol. Fertil. Soils* **39,** 65–73 (2003).
140. Sedia, E. G. & Ehrenfeld, J. G. Differential effects of lichens and mosses on soil enzyme activity and litter decomposition. *Biol. Fertil. Soils* **43,** 177–189 (2006).
141. Senwo, Z. N., Ranatunga, T. D., Tazisong, I. A., Taylor, R. W. & He, Z. Phosphatase activity of Ultisols and relationship to soil fertility indices. *J. Food Agric. Environ.* **5,** 262 (2007).
142. Shange, R. S., Ankumah, R. O., Ibekwe, A. M., Zabawa, R. & Dowd, S. E. Distinct soil bacterial communities revealed under a diversely managed agroecosystem. *PLoS One* **7,** (2012).
143. Shi, X.-M. *et al.* Grazing exclusion decreases soil organic C storage at an alpine grassland of the Qinghai–Tibetan Plateau. *Ecol. Eng.* **57,** 183–187 (2013).
144. Shillam, L., Hopkins, D. W., Badalucco, L. & Laudicina, V. A. Structural diversity and enzyme activity of volcanic soils at different stages of development and response to experimental disturbance. *Soil Biol. Biochem.* **40,** 2182–2185 (2008).
145. Sicardi, M., García-Préchac, F. & Frioni, L. Soil microbial indicators sensitive to land use conversion from pastures

to commercial Eucalyptus grandis (Hill ex Maiden) plantations in Uruguay. *Appl. Soil Ecol.* **27,** 125–133 (2004).

146. Sidari, M., Ronzello, G., Vecchio, G. & Muscolo, A. Influence of slope aspects on soil chemical and biochemical properties in a Pinus laricio forest ecosystem of Aspromonte (Southern Italy). *Eur. J. Soil Biol.* **44,** 364–372 (2008).

147. da Silva, L. G. *et al.* Atributos físicos, químicos e biológicos de um Latossolo de cerrado em plantio de espécies florestais. *Pesqui. agropecuária Bras.* **44,** 613–620 (2010).

148. Singh, K., Singh, B. & Singh, R. R. Changes in physico-chemical, microbial and enzymatic activities during restoration of degraded sodic land: Ecological suitability of mixed forest over monoculture plantation. *Catena* **96,** 57–67 (2012).

149. Singh, S. K., Rai, J. P. N. & Singh, A. Influence of prevailing disturbances on soil biology and biochemistry of montane habitats at Nanda Devi Biosphere Reserve (NDBR), India during wet and dry seasons. *Geoderma* **162,** 296–302 (2011).

150. Souza-Alonso, P., Lorenzo, P., Rubido-Bará, M. & González, L. Effectiveness of management strategies in Acacia dealbata Link invasion, native vegetation and soil microbial community responses. *For. Ecol. Manage.* **304,** 464–472 (2013).

151. Staddon, W. J., Duchesne, L. C. & Trevors, J. T. Acid phosphatase, alkaline phosphatase and arylsulfatase activities in soils from a jack pine (Pinus banksiana Lamb.) ecosystem after clear-cutting, prescribed burning, and scarification. *Biol. Fertil. Soils* **27,** 1–4 (1998).

152. Sun, X., Gao, C. & Guo, L. D. Changes in soil microbial community and enzyme activity along an exotic plant Eupatorium adenophorum invasion in a Chinese secondary forest. *Chinese Sci. Bull.* **58,** 4101–4108 (2013).

153. Sun, S. Q., He, G., Wu, Y. H., Zhou, J. & Yu, D. Starch and nutrient contents are key for mosses adapting to different succession stages along a receding glacier. *Polish J. Ecol.* **61,** 233–239 (2013).

154. Tan, X., Chang, S. X. & Kabzems, R. Soil compaction and forest floor removal reduced microbial biomass and enzyme activities in a boreal aspen forest soil. *Biol. Fertil. Soils* **44,** 471–479 (2008).

155. Turner, B. L. & Haygarth, P. M. Phosphatase activity in temperate pasture soils: Potential regulation of labile organic phosphorus turnover by phosphodiesterase activity. *Sci. Total Environ.* **344,** 27–36 (2005).

156. Turner, B. L., Baxter, R. & Whitton, B. A. Seasonal phosphatase activity in three characteristic soils of the English uplands polluted by long-term atmospheric nitrogen deposition. *Environ. Pollut.* **120,** 313–317 (2002).

157. Turrión, M. B., Schneider, K. & Gallardo, J. F. Soil P availability along a catena located at the Sierra de Gata Mountains, Western Central Spain. *For. Ecol. Manage.* **255,** 3254–3262 (2008).

158. Ushio, M., Kitayama, K. & Balser, T. C. Tree species effects on soil enzyme activities through effects on soil physicochemical and microbial properties in a tropical montane forest on Mt. Kinabalu, Borneo. *Pedobiologia (Jena).* **53,** 227–233 (2010).

159. Vallejo, V. E. *et al.* Effect of land management and Prosopis juliflora (Sw.) DC trees on soil microbial community and enzymatic activities in intensive silvopastoral systems of Colombia. *Agric. Ecosyst. Environ.* **150,** 139–148 (2012).

160. Vallejo, G., Ballare, C., Lino Barañao, J., Beato, M. & Saragueta, P. Progestin activation of nongenomic pathways via cross talk of progesterone receptor with estrogen receptor β induces proliferation of endometrial stromal cells. *Mol. Endocrinol.* **19,** 3023–3037 (2005).

161. Vallejo, V. E., Roldan, F. & Dick, R. P. Soil enzymatic activities and microbial biomass in an integrated agroforestry chronosequence compared to monoculture and a native forest of Colombia. *Biol. Fertil. Soils* **46,** 577–587 (2010).

162. Vance, N. C. & Entry, J. A. Soil properties important to the restoration of a Shasta red fir barrens in the Siskiyou Mountains. *For. Ecol. Manage.* **138,** 427–434 (2000).

163. Vasconcellos, R. L. F. *et al.* Microbiological indicators of soil quality in a riparian forest recovery gradient. *Ecol. Eng.* **53,** 313–320 (2013).

164. Viñegla, B., García-Ruiz, R., Liétor, J., Ochoa, V. & Carreira, J. A. Soil phosphorus availability and transformation rates in relictic pinsapo fir forests from southern Spain. *Biogeochemistry* **78,** 151–172 (2006).

165. Wang, Q. K., Wang, S. L. & Liu, Y. X. Responses to N and P fertilization in a young Eucalyptus dunnii plantation: Microbial properties, enzyme activities and dissolved organic matter. *Appl. Soil Ecol.* **40,** 484–490 (2008).

166. Wang, Q., Bao, Y., Liu, X. & Du, G. Spatio-temporal dynamics of arbuscular mycorrhizal fungi associated with glomalin-related soil protein and soil enzymes in different managed semiarid steppes. *Mycorrhiza* **24,** 525–538 (2014).

167. Wang, Q., Xiao, F., He, T. & Wang, S. Responses of labile soil organic carbon and enzyme activity in mineral soils to forest conversion in the subtropics. *Ann. For. Sci.* **70,** 579–587 (2013).

168. Wang, Q., Xiao, F., Zhang, F. & Wang, S. Labile soil organic carbon and microbial activity in three subtropical plantations. *Forestry* **86,** 569–574 (2013).

169. Wang, R. *et al.* Coupled response of soil carbon and nitrogen pools and enzyme activities to nitrogen and water addition in a semi-arid grassland of Inner Mongolia. *Plant Soil* **381,** 323–336 (2014).

170. Wick, B., Kühne, R. F., Vielhauer, K. & Vlek, P. L. G. Temporal variability of selected soil microbiological and biochemical indicators under different soil quality conditions in south-western Nigeria. *Biol. Fertil. Soils* **35,** 155–167 (2002).

171. Yadav, R. S., Yadav, B. L., Chhipa, B. R., Dhyani, S. K. & Ram, M. Soil biological properties under different tree

based traditional agroforestry systems in a semi-arid region of Rajasthan, India. *Agrofor. Syst.* **81,** 195–202 (2011).
172. Yang, K., Zhu, J. J., Yan, Q. L. & Sun, O. J. Changes in soil P chemistry as affected by conversion of natural secondary forests to larch plantations. *For. Ecol. Manage.* **260,** 422–428 (2010).
173. Yang, Q. *et al.* Structure and function of soil microbial community in artificially planted Sonneratia apetala and S. caseolaris forests at different stand ages in Shenzhen Bay, China. *Mar. Pollut. Bull.* **85,** 754–763 (2014).
174. Zhang, C., Liu, G., Xue, S. & Song, Z. Rhizosphere soil microbial activity under different vegetation types on the Loess Plateau, China. *Geoderma* **161,** 115–125 (2011).
175. Zhang, C., Liu, G., Xue, S. & Zhang, C. Rhizosphere soil microbial properties on abandoned croplands in the Loess Plateau, China during vegetation succession. *Eur. J. Soil Biol.* **50,** 127–136 (2012).
176. Zhang, Z. S. *et al.* Soil oxidases recovered faster than hydrolases in a 50-year chronosequence of desert revegetation. *Plant Soil* **358,** 275–287 (2012).
177. Zhao, Q., Zhou, L., Zheng, X., Wang, Y. & Lu, J. Study on enzymatic activities and behaviors of heavy metal in sediment-plant at muddy tidal flat in Yangtze Estuary. *Environ. Earth Sci.* **73,** 3207–3216 (2015).
178. Zhou, X. *et al.* Warming and increased precipitation have differential effects on soil extracellular enzyme activities in a temperate grassland. *Sci. Total Environ.* **444,** 552–558 (2013).
179. Zhou, X., Zhang, Y. & Downing, A. Non-linear response of microbial activity across a gradient of nitrogen addition to a soil from the Gurbantunggut Desert, northwestern China. *Soil Biol. Biochem.* **47,** 67–77 (2012).
180. Zhu, F., Yoh, M., Gilliam, F. S., Lu, X. & Mo, J. Nutrient limitation in three lowland tropical forests in southern China receiving high nitrogen deposition: Insights from fine root responses to nutrient additions. *PLoS One* **8,** 1–8 (2013).
181. Zimmermann, S. & Frey, B. Soil respiration and microbial properties in an acid forest soil: effects of wood ash. *Soil Biol. Biochem.* **34,** 1727–1737 (2002).
182. Zornoza, R. *et al.* Soil properties under natural forest in the Alicante Province of Spain. *Geoderma* **142,** 334–341 (2007).
183. Han, J., Jung, J., Hyun, S., Park, H. & Park, W. Effects of nutritional input and diesel contamination on soil enzyme activities and microbial communities in Antarctic soils. *J. Microbiol.* **50,** 916–924 (2012).
